# AF4 uses the SL1 components of RNAP1 machinery to initiate MLL fusion- and AEP-dependent transcription

**DOI:** 10.1038/ncomms9869

**Published:** 2015-11-23

**Authors:** Hiroshi Okuda, Akinori Kanai, Shinji Ito, Hirotaka Matsui, Akihiko Yokoyama

**Affiliations:** 1 Laboratory for Malignancy Control Research, Kyoto University Graduate School of Medicine, 53 Kawahara-cho, Shogoin, Sakyo-ku, Kyoto 606-8501, Japan; 2 Department of Molecular Oncology and Leukemia Program Project, Research Institute for Radiation Biology and Medicine, Hiroshima University, 1-2-3 Kasumi, Minami-ku, Hiroshima 734-8553, Japan; 3 Medical Research Support Center, Kyoto University Graduate School of Medicine, Yoshidakonoe-cho, Sakyo-ku, Kyoto 606-8501, Japan; 4 Present address: Department of Molecular Laboratory Medicine, Graduate School of Medical Sciences, Kumamoto University, 1-1-1 Honjo, Chuo-ku, Kumamoto 860-8556, Japan

## Abstract

Gene rearrangements generate *MLL* fusion genes, which can lead to aggressive leukemia. In most cases, *MLL* fuses with a gene encoding a component of the AEP (AF4 family/ENL family/P-TEFb) coactivator complex. MLL–AEP fusion proteins constitutively activate their target genes to immortalize haematopoietic progenitors. Here we show that AEP and MLL–AEP fusion proteins activate transcription through selectivity factor 1 (SL1), a core component of the pre-initiation complex (PIC) of RNA polymerase I (RNAP1). The pSER domain of AF4 family proteins associates with SL1 on chromatin and loads TATA-binding protein (TBP) onto the promoter to initiate RNA polymerase II (RNAP2)-dependent transcription. These results reveal a previously unknown transcription initiation mechanism involving AEP and a role for SL1 as a TBP-loading factor in RNAP2-dependent gene activation.

## Abstract (web summary)

Protein fusions between MLL and AEP (AF4 family/ENL family/P-TEFb) constitutively activate their target genes to immortalize hematopoietic progenitors. Here, Okuda *et al*. show that MLL-AEP binds SL1, a component of the pre-initiation complex of RNA polymerase (RNAP) I, to initiate RNAP II dependent transcription.

## Graphical abstract

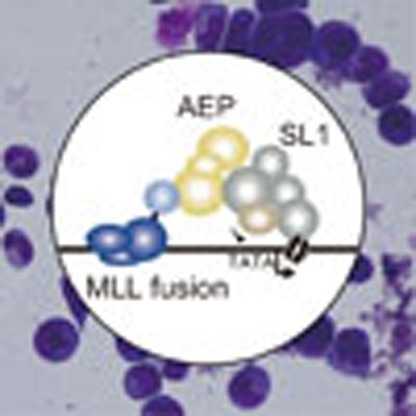

Chromosomal translocations generate a variety of *MLL* (also known as *KMT2A*, *MLL1*, *HRX* and *ALL-1*) fusion genes, which cause acute leukemia in myeloid and lymphoid lineages^1^. Although >70 different fusion partners have been identified^2^, the majority of leukemia cases are caused by the chimeric genes formed by *MLL* and a gene encoding a component of the AEP (AF4 family/ENL family/P-TEFb) coactivator complex^3^. The AEP complex comprises AF4 family proteins (for example, AF4 and AF5Q31), ENL family proteins (for example, ENL and AF9) and the P-TEFb elongation factor. Similar, if not identical, complexes have been identified and shown to play important roles in various biological processes (for example, heat shock response and transcription of the HIV viral genome)^4^,^5^,^6^,^7^. AEP associates with RNA polymerase II (RNAP2)-specific factors, including the polymerase II-associated factor 1 complex^5^ and the mediator complex^8^, and thus appears to be closely linked to RNAP2-dependent transcription. MLL–AEP fusion proteins constitutively activate their target genes by recruiting AEP components to the target chromatin, whereas wild-type MLL recruits AEP in a context-dependent manner^3^. In the haematopoietic lineage, MLL fusion proteins aberrantly activate a subset of genes implicated in the haematopoietic stem cell programme, such as *HOXA9* and *MEIS1* (ref. 9). Constitutive expression of these genes in haematopoietic progenitors has been shown to induce leukemia in a mouse model^10^, suggesting that a gain-of-function mechanism underlies the development of MLL leukemia. MLL fusion proteins form a complex with MENIN and LEDGF, and the MLL fusion protein complex directly binds to target chromatin through the PWWP domain of LEDGF and the CXXC domain of MLL^11^,^12^,^13^. The PWWP domain recognizes di-/trimethylated histone H3 lysine 36, which normally associates with transcriptionally active regions^14^,^15^. The CXXC domain specifically binds to non-methylated CpGs, which are enriched in active promoters^16^. Consequently, MLL–AEP fusion proteins target previously active CpG-rich promoters, where they recruit AEP components to activate transcription. As AEP contains the P-TEFb elongation factor, it has been suggested that MLL–AEP fusion proteins mainly activate transcription by releasing RNAP2 from promoter-proximal pausing^17^. However, it remains largely unknown how MLL–AEP fusion proteins activate their target genes.

Here we report that a serine-rich domain in AF4 family proteins, termed pSER, is an essential functional component of MLL–AEP fusion-dependent gene activation and leukemic transformation. Through biochemical purification, we identified selectivity factor 1 (SL1) as a novel factor associated with the pSER domain. SL1, comprising TATA-binding protein (TBP) and four TATA box-binding protein-associated factors (TAF_I_s; TAF1A/TAF_I_48, TAF1B/TAF_I_63, TAF1C/TAF_I_110 and TAF1D/TAF_I_41), is a core component of the pre-initiation complex (PIC) of RNA polymerase I (RNAP1; refs 18, 19, 20, 21). In the presence of upstream binding factor (UBF), SL1 forms a PIC on the promoters of ribosomal RNA genes to drive RNAP1-dependent transcription^22^. However, it is unknown whether SL1 plays a role in RNAP2-dependent transcription. Our results indicate that the AEP coactivator complex facilitates the initiation of RNAP2-dependent transcription via SL1 activity by loading TBP onto the TATA element. MLL–AEP fusion proteins use this TBP-loading function to activate transcription in leukemic transformation, whereas the wild-type AEP complex activates gene expression in the same manner under physiological conditions.

## Results

### The pSER domain drives myeloid transformation

**Figure 1:**
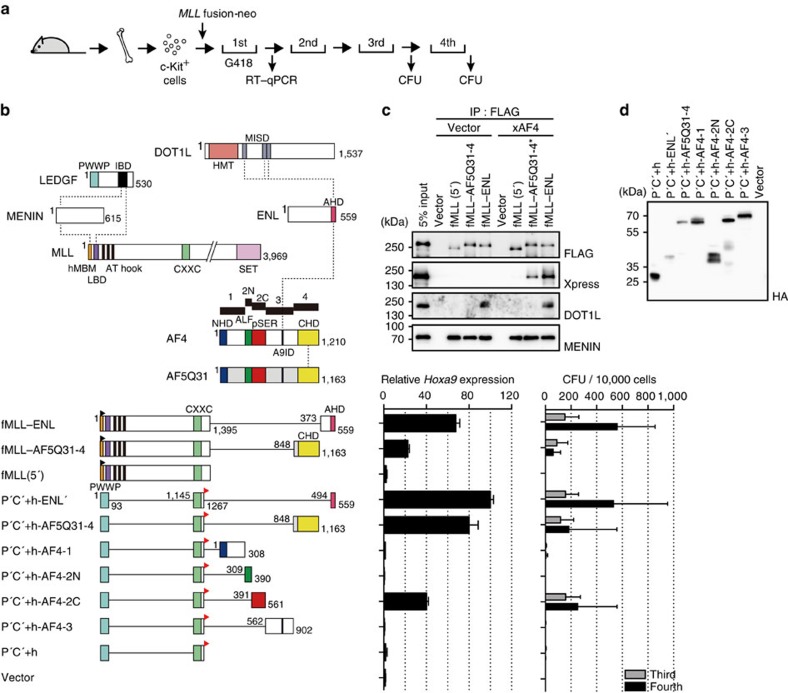
MLL–AEP transforms myeloid progenitors through the pSER domain. (**a**) Schema of the myeloid progenitor transformation assay. Various constructs for MLL fusion genes cloned into the pMSCV-neo vector were used for gene transduction. (**b**) Transforming ability of various MLL–AEP fusion proteins. AF4 and AF5Q31 were subdivided into five regions designated 1, 2N, 2C, 3 and 4. P′, the minimum structure of the PWWP domain of LEDGF; C′+, the minimum structure of the CXXC domain of MLL and its C-terminal region; ENL′, the minimum structure of the AHD; f, the FLAG epitope (black flag); h, the haemagglutinin (HA) epitope (red flag). *Hoxa9* expression normalized to *Gapdh* is shown as the relative value of P′C′+h-ENL′ (arbitrarily set at 100%) with error bars (s.d. of PCR triplicates). The number of colony-forming units (CFUs) at the third and fourth rounds of replating is shown with error bars (s.d. from >3 independent experiments). AHD, ANC homology domain; ALF, AF4/LAF4/FMR2 homology domain; A9ID, AF9 interaction domain; CHD, C-terminal homology domain; CXXC, CXXC domain; hMBM, high affinity MENIN-binding motif; HMT, histon methyltransferase catalytic domain; IBD, integrase-binding domain; LBD, LEDGF-binding domain; MISD, minimum interaction site of DOT1L; NHD, N-terminal homology domain; pSER, homology domain containing a high number of serines (poly-serine); PWWP, PWWP domain; SET, SET domain. (**c**) AF4 binds MLL–ENL and MLL–AF5Q31-4. AF4 tagged with an Xpress epitope (denoted by x) was co-expressed with FLAG-tagged MLL–AEP fusion proteins in 293T cells and subjected to IP–WB. The *MLL* fusion genes and the *AF4* gene were cloned into pCMV5 vector and the pcDNA4 HisMax vector, respectively, and used for transient expression. The sample shown in the input lane is indicated by an asterisk. (**d**) Protein expression of the various MLL mutants. The expression of various P′C′+ mutants in the virus packaging cells was visualized using an anti-HA antibody.

In *ex vivo* conditions, MLL fusion proteins transform myeloid progenitors by constitutively activating haematopoietic stem cell programme genes such as *Hoxa9* (ref. 23). As transformation leads to the immortalization of myeloid progenitors, it is a critical event in leukemogenesis induced by MLL fusion proteins^24^. Their transforming properties are evidenced by sustained expression of *Hoxa9* in the first round colonies and vigorous colony-forming activities in the third and fourth rounds of replating in myeloid progenitor transformation assays (Fig. 1a,b). The minimal functional domains of the fusion partner portions of MLL–ENL and MLL–AF5Q31 required for transformation are the ANC1 homology domain (AHD) of ENL (also known as MLLT1)^11^,^25^ and the carboxy-terminal homology domain (CHD) of AF5Q31 (also known as AFF4), respectively^3^ (Fig. 1b). Immunoprecipitation (IP) followed by western blotting (WB) showed that both of the fusion partner portions serve as a binding platform for AF4 (also known as AFF1). The ENL portion also associated with DOT1L, another ENL-associated factor^7^ implicated in MLL fusion-dependent leukemogenesis^26^,^27^,^28^,^29^,^30^. On the other hand, MLL–AF5Q31-4 did not pull down DOT1L, indicating that direct recruitment of DOT1L is not critical for transformation (Fig. 1c). Removal of the AF4-binding platform resulted in loss of transforming activity^3^ (Fig. 1b). Thus, recruitment of AF4 appears to be essential for MLL–AEP fusion-dependent gene activation and transformation. With that in mind, we inferred that one or more functional domains in AF4, besides the CHD, are responsible for MLL–AEP fusion-dependent gene activation and transformation. Recently, we identified the minimum targeting module of MLL–AEP fusion proteins that is sufficient for the recognition of target chromatin. The module comprises the PWWP domain and the CXXC domain^11^ (Fig. 1b). An artificial gene in which the minimum targeting module was fused to the AHD of ENL or to the CHD of AF5Q31 activated *Hoxa9* and immortalized myeloid progenitors (Fig. 1b). To investigate the role of the functional domains of AF4 in MLL fusion-dependent transformation, we constructed a series of mutants in which the minimum targeting module was tethered to the subdivided domains of AF4, termed AF4-1, AF4-2N, AF4-2C and AF4-3 (Fig. 1b,d), and examined their transforming ability. Of the four AF4 domains, only the AF4-2C domain, which encompasses the evolutionarily conserved pSER domain, exhibited transforming abilities (Fig. 1b). These results suggest that the pSER domain of AF4 mediates the transformation of myeloid progenitors induced by MLL–AEP fusion proteins.

### The pSER domain associates with SL1 on chromatin

**Figure 2:**
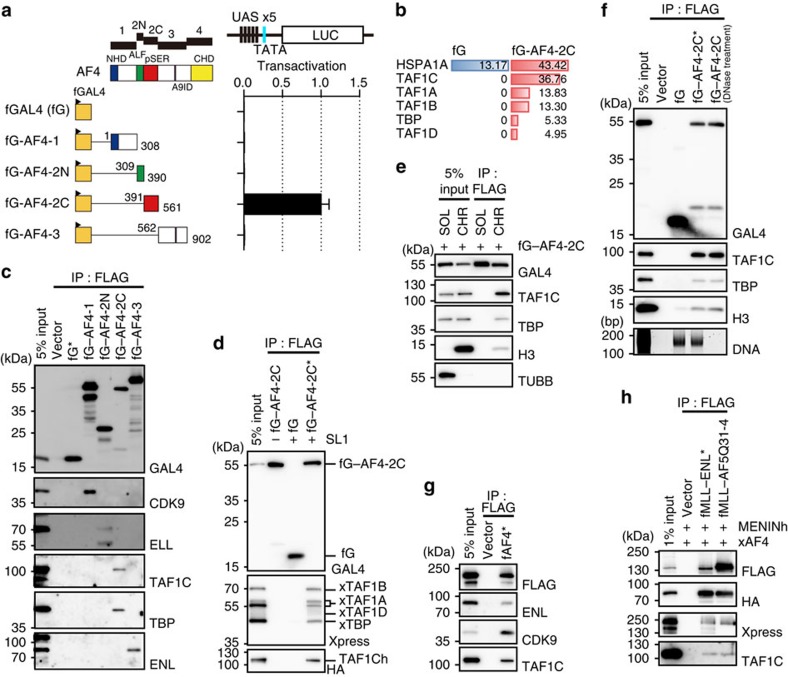
The pSER domain associates with SL1 on chromatin and activates transcription. (**a**) Transactivation activities of the AF4 subdomains. The schematic structures of various FLAG-tagged GAL4 fusion proteins are shown. Various GAL4 fusion genes were cloned into pCMV5 vector and used for transient expression. fG, FLAG-tagged GAL4 DNA-binding domain. 293T cells were transiently transfected with the expression vectors for various GAL4 fusion proteins with the pFR–LUC and pRL–TK reporter plasmids. Promoter activity was assessed with the dual luciferase reporter assay. The transcription activation activity, normalized to the RL activity, is shown with error bars (s.d. from triplicates) relative to the value of fG–AF4-2C (arbitrarily set at 1). UAS, upstream activation sequence. (**b**) The protein scores of SL1 components identified by mass spectrometry as co-precipitates with fG–AF4-2C. (**c**) Factors associated with various AF4 subdomains. Various FLAG-tagged GAL4–AF4 fusion proteins were expressed in 293T cells and subjected to fanChIP–WB. The sample shown in the input lane is indicated by an asterisk. (**d**) Association of the SL1 complex with the pSER domain. All SL1 components were co-expressed with fG-AF4-2C in 293T cells and subjected to fanChIP–WB. The pcDNA4 HisMax-TAF1A/B/D and -TBP vectors and the pCMV5-TAF1C-HA vector were used for transient expression of Xpress-tagged or HA-tagged SL1 components. (**e**) Chromatin-specific association of SL1 with the pSER domain. IP–WB analyses were performed with the chromatin fraction (CHR) or the chromatin-unbound soluble faction (SOL) from 293T cells expressing fG-AF4-2C. (**f**) DNA-independent association of SL1 with the pSER domain. FanChIP analysis, as described in **c**, was performed, followed by DNaseI treatment. (**g**) Association of SL1 with AEP. FLAG-tagged AF4 was transiently expressed in 293T cells and subjected to fanChIP–WB. The pBICEP2–AF4 vector was used for transient expression. (**h**) Association of SL1 with MLL–AEP fusion proteins. FLAG-tagged MLL–AEP fusion proteins were co-expressed with Xpress-tagged AF4 (xAF4) and HA-tagged MENIN (MENINh) in 293T cells and subjected to fanChIP–WB. The pCMV5-MLL fusion vectors, the pcDNA 3.1 hygro (+)-MEN1-HA vector and the pcDNA4 HisMax-AF4 vector were used for transient expression.

Next, we characterized the transcriptional properties of each AF4 domain. To this end, we generated a series of constructs in which a GAL4 DNA-binding domain was fused to each subdivided domain of AF4. Transactivation assays using a GAL4-responsive reporter showed that the FLAG-tagged GAL4–AF4-2C fusion protein (fG–AF4-2C) had substantial transactivation activity (Fig. 2a), consistent with previous reports^3^,^31^,^32^. As transcription takes place on chromatin *in vivo*, we inferred that the pSER domain associated with cofactors on chromatin to activate transcription. To purify the chromatin protein complex, we used the fractionation-assisted native chromatin IP (fanChIP) method, which we previously established^11^. In this method, chromatin-unbound materials are removed by cytoskeleton buffer extraction, and chromatin and chromatin-bound materials are solubilized by micrococcal nuclease (MNase) digestion followed by exposure to detergent (Supplementary Fig. 1a). The factors that associated specifically with fG–AF4-2C on chromatin were copurified from the chromatin fraction by affinity purification using an anti-FLAG antibody (Supplementary Fig. 1b). Mass spectrometry analysis of the purified materials showed that all components of SL1 bound to the pSER domain (Fig. 2b). FanChIP analysis followed by WB (fanChIP–WB) confirmed that the pSER domain associated specifically with endogenous TAF1C and TBP, but not with TFIIB, TAF1 (TAF_II_p250), RRN3 or RNAP1 (POLR1A; Supplementary Fig. 1c), whereas the other domains exhibited their own cofactor binding properties (for example, AF4-1-bound CDK9 (a component of P-TEFb) and AF4-2N-bound Eleven-nineteen lysine-rich leukemia protein (ELL); Fig. 2c). The pSER domain pulled down all the components of SL1 that were exogenously expressed, either individually (Supplementary Fig. 1d) or simultaneously (Fig. 2d). This interaction occurred only on chromatin (Fig. 2e) but was not dependent on DNA (Fig. 2f). Wild-type AF4 also pulled down TAF1C, along with ENL and CDK9 (Fig. 2g). Moreover, MLL–ENL and MLL–AF5Q31-4 pulled down TAF1C when co-expressed with MENIN and AF4 (Fig. 2h). Hence, both AEP and the MLL–AEP fusion proteins specifically associate with SL1 on chromatin.

### MLL–ENL co-localizes with TAF1C at the target promoters

**Figure 3:**
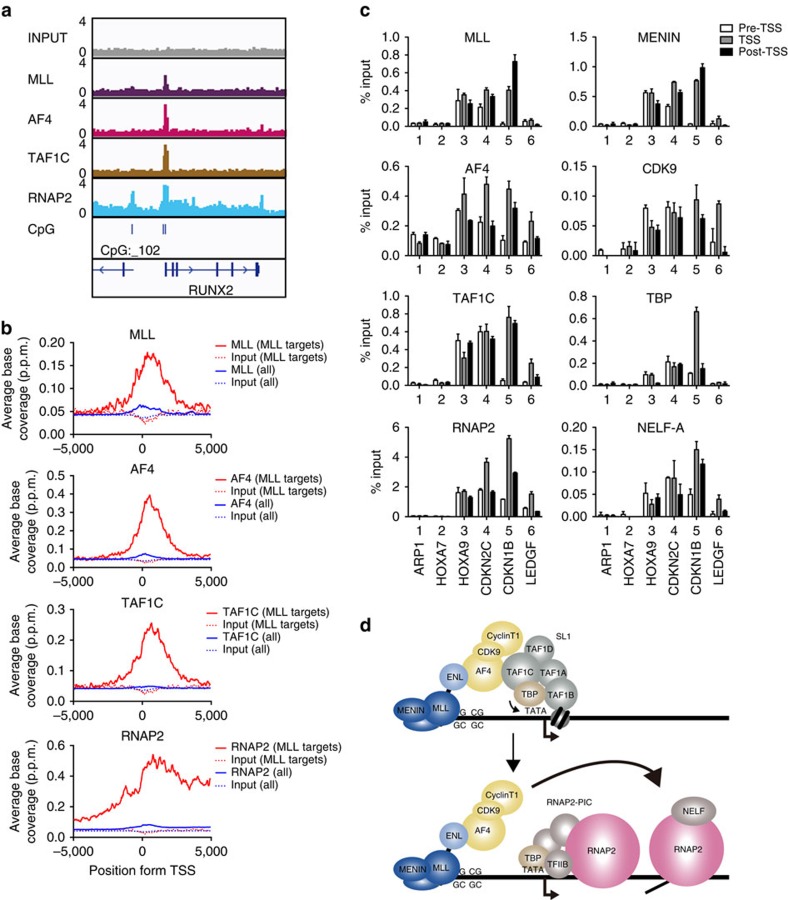
MLL–ENL co-localizes with TAF1C at target promoters. (**a**) Localization of MLL–ENL, AF4, TAF1C and RNAP2 at the RUNX2 locus in HB1119 cells. Chromatin from HB1119 cells was used in fanChIP-seq analysis with the indicated antibodies. The ChIP signals were visualized using Integrative Genome Viewer (Broad Institute). (**b**) The average distribution of MLL, AF4, TAF1C and RNAP2 at the MLL-occupied loci (red line). The average distribution of each protein at all transcription start sites (TSSs; blue line) and of the input DNA (broken lines) is included for comparison. The *y* axis indicates the ChIP-seq tag count (p.p.m.) in 100-bp increments. (**c**) Localization of MLL, MENIN, AF4, CDK9, TAF1C, TBP, RNAP2 and NELF-A at various loci in HB1119 cells. The genomic localization of each protein was determined with fanChIP–qPCR. The precipitated DNA was analysed using specific probes for the pre-TSS (−1.0 to −0.5 kb from the TSS), TSS (0 to +0.5 kb from the TSS) and post-TSS (+1.0 to +1.5 kb from the TSS) regions of the indicated genes. The ChIP signals were expressed as the per cent input with error bars (s.d. of PCR triplicates). (**d**) A model for the cooperative transactivation of MLL target genes by SL1 and the RNAP2 transcriptional machinery. PIC, pre-initiation complex.

To examine whether MLL–AEP fusion proteins co-localize with SL1 at target promoters, we performed a fanChIP assay followed by deep sequencing (fanChIP-seq) with HB1119 cells, which harbour a t(11;19) translocation and therefore express the MLL–ENL protein. From the enriched ChIP signals obtained using an anti-MLL antibody (Fig. 3a and Supplementary Fig. 2a), we identified a representative set of MLL target genes (112 genes), which included many previously identified direct MLL target genes such as *HOXA9*, *MEIS1*, *RUNX2*, *CDKN1B* and *CDKN2C*^3^,^33^ (Supplementary Table 1). The average distribution of the MLL proteins at the MLL target promoters suggested that MLL–ENL associated with chromatin near the transcription start sites (Fig. 3b). ChIP signals for AF4 and TAF1C were also observed at the MLL target promoters (Fig. 3a,b and Supplementary Fig. 2a). The signal intensity of TAF1C was highly correlated with those of MLL and AF4 at the promoter proximal regions of the MLL target loci (Supplementary Fig. 2b). This was confirmed by the results of a fanChIP assay followed by quantitative PCR (qPCR) analysis (fanChIP–qPCR), performed for several MLL target loci (Fig. 3c). FanChIP–qPCR analysis using anti-MENIN, anti-CDK9 and anti-TBP antibodies demonstrated that the MLL–ENL/AF4/SL1 complex formed at the MLL target promoters. RNAP2 and its cofactors, such as NELF, also localized at the MLL target promoters (Fig. 3c). Expression of these genes was sensitive to α-amanitin treatment (Supplementary Fig. 2c), suggesting that RNAP2-dependent transcription occurs at MLL–ENL target genes. These results indicate that SL1 facilitates, rather than inhibits, RNAP2-dependent transcription at MLL target genes (Fig. 3d).

### TAF1C is required for AEP-dependent gene activation

**Figure 4:**
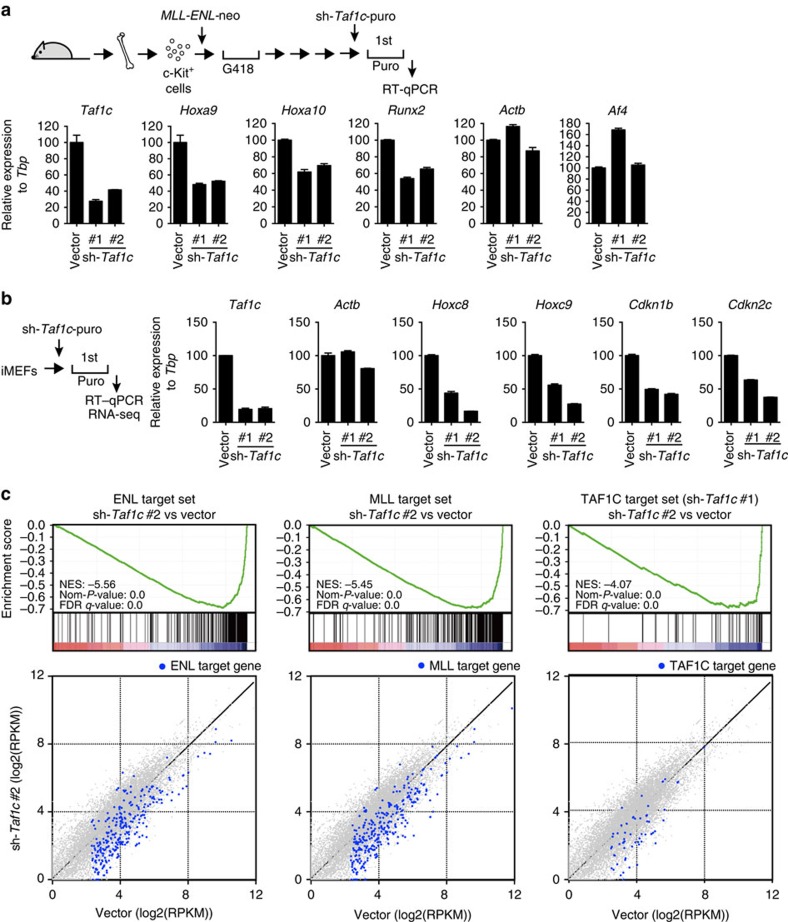
TAF1C is required for gene activation by MLL–ENL and AEP. (**a**) The expression of MLL–ENL target genes (*Hoxa9*, *Hoxa10* and *Runx2*) after knockdown of *Taf1c* with two different shRNAs in MLL–ENL-transformed cells. The expression level normalized to *Tbp* is shown relative to the value of the vector control (arbitrarily set at 100%) with error bars (s.d. of PCR triplicates). Puro, puromycin. (**b**) Effect of *Taf1c* knockdown on AEP-dependent gene activation. *Taf1c* was knocked down with two different shRNAs in iMEFs. The expression of AEP target genes (*Hoxc8*, *Hoxc9*, *Cdkn1b* and *Cdkn2c*) was analysed with reverse transcriptase (RT)–qPCR as described in **a**. (**c**) Gene set enrichment analysis (GSEA) of the expression profiles of iMEFs after knockdown of *Taf1c* (#1 and #2), *Enl* and *Mll*. Genes that exhibited a greater than threefold decrease on knockdown in RNA sequencing (RNA-seq) were defined as target genes (Supplementary Fig. 3c,d). The target gene sets of ENL, MLL and TAF1C (shRNA#1) were downregulated by knockdown of *Taf1c* with shRNA#2. Expression levels of the target genes were also shown by scatter plots. Target genes of ENL, MLL and TAF1C are highlighted in blue. FDR, false discovery rate; NES, normalized enrichment score; Nom-*P*-value, nominal *P*-value; RPKM, reads per kilobase of exon per million mapped reads.

To examine whether SL1 is required for MLL–ENL-dependent gene activation, we knocked down *Taf1c* in MLL–ENL-transformed cells by using two different short hairpin RNAs (shRNAs). Knockdown of *Taf1c* decreased the expression of MLL–ENL target genes (*Hoxa9*, *Hoxa10* and *Runx2*; Fig. 4a). These results suggest that TAF1C is an essential cofactor for MLL–AEP fusion-dependent gene activation.

Next, we tested whether SL1 is required for AEP-dependent transcription in a non-oncogenic context. *Taf1c* was knocked down in immortalized mouse embryonic fibroblasts (iMEFs). As wild-type MLL collaborates with AEP at many target genes^3^, the expressions of *Hoxc8*, *Hoxc9*, *Cdkn1b* and *Cdkn2c* are dependent on both ENL and MLL in iMEFs (Supplementary Fig. 3a,b). shRNAs against *Taf1c* decreased the expression of the MLL/AEP target genes (Fig. 4b). Moreover, messenger RNA sequencing analysis showed that knockdown of *Taf1c* globally downregulated ENL and MLL target genes in iMEFs (Fig. 4c and Supplementary Fig. 3c–g). These results indicate that SL1 is required for gene activation mediated by the MLL/AEP axis.

### Unique mode of transcription initiation by the pSER domain

**Figure 5:**
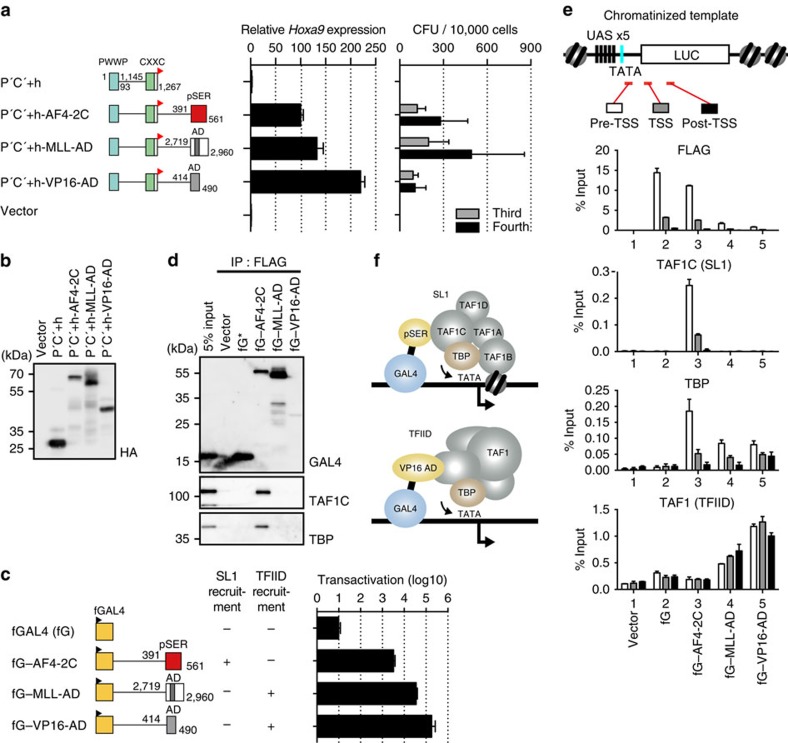
Transcription initiation by the pSER domain confers transforming properties. (**a**) Transforming abilities of P′C′+ mutants fused with various ADs. *Hoxa9* expression normalized to *Gapdh* is shown relative to the value of P′C′+h-AF4-2C (arbitrarily set at 100%) with error bars (s.d. of PCR triplicates). The colony-forming abilities were analysed as described for Fig. 1b. (**b**) Protein expression of the P′C′+ mutants in the virus packaging cells. (**c**) Transcription activation activity of various GAL4–AD fusion proteins on the chromatinized GAL4-responsive promoter. 293T–LUC cells were transiently transfected with the pCMV5 expression vectors for various FLAG-tagged GAL4 fusion proteins and the pRL–TK reporter plasmid. Promoter activity was assessed with the dual luciferase reporter assay. The normalized transcription activation activity is shown in the logarithmic scale relative to the value of fG (arbitrarily set at 10). (**d**) Association of SL1 components with various transcriptional activation domains. FLAG-tagged GAL4–AD fusion proteins were expressed in 293T–LUC cells and subjected to fanChIP–WB. The sample shown in the input lane is indicated by an asterisk. (**e**) Localization of FLAG-tagged GAL4–AD fusion proteins, TAF1C, TBP and TAF1 on the chromatinized luciferase promoter. Each FLAG-tagged GAL4–AD fusion protein was expressed in 293T–LUC cells carrying the FR–LUC reporter cassette inserted in the genome and subjected to fanChIP–qPCR with the indicated antibodies. Precipitated DNA was analysed with qPCR, using probes specific to the pre-transcription start site (TSS), TSS and post-TSS regions. (**f**) Models of alternative RNAP2-dependent transcription initiation by SL1 and TFIID.

To examine whether the initiation of RNAP2-dependent transcription by the pSER domain is responsible for MLL–AEP fusion-dependent transformation, we generated artificial genes in which the minimum targeting module of MLL was fused to various transactivation domains (ADs) that have been shown to initiate RNAP2-dependent transcription. We tested their transforming ability in myeloid progenitor transforming assays (Fig. 5a,b). The AD of VP16 recruits mediators, the basic transcriptional machinery and CBP/p300 coactivators to initiate RNAP2-dependent transcription^34^,^35^,^36^,^37^. The AD of MLL also recruits CBP/p300 coactivators (Supplementary Fig. 4) to activate transcription in transactivation assays^38^,^39^. Both ADs were able to functionally substitute for the pSER domain of AF4 in the myeloid progenitor transformation assay (Fig. 5a,b). Hence, the ability to initiate RNAP2-dependent transcription appears to be an essential function of the pSER domain in MLL–AEP fusion-dependent transformation. Next, we generated constructs in which a GAL4 DNA-binding domain was fused to the same ADs (Fig. 5c). FanChIP–WB analysis of the GAL4 fusion proteins showed that only the pSER domain formed a complex with SL1 (Fig. 5d). To analyse transcription activation in a chromatin context, we established a 293T cell line in which the GAL4-responsive reporter cassette was inserted into the genome. Transactivation assays using this cell line showed that the pSER domain retained its transactivation activity in a chromatin context, which was the weakest of the three ADs (Fig. 5c) and was susceptible to α-amanitin treatment (Supplementary Fig. 4b). FanChIP–qPCR analysis showed that both TAF1C and TBP were efficiently recruited to the promoter through the pSER domain, whereas the ADs of MLL and VP16 recruited TBP, but not TAF1C, to the promoter (Fig. 5e). TAF1, a component of the TFIID complex^40^, was recruited to the promoter by the ADs of MLL and VP16 but not by the pSER domain (Fig. 5e). These results suggest that the pSER domain uses SL1 exclusively as the primary TBP recruiting factor to activate transcription initiation, whereas the ADs of MLL and VP16 use TFIID for TBP recruitment (Fig. 5f).

### The pSER domain loads TBP onto the TATA element via SL1

**Figure 6:**
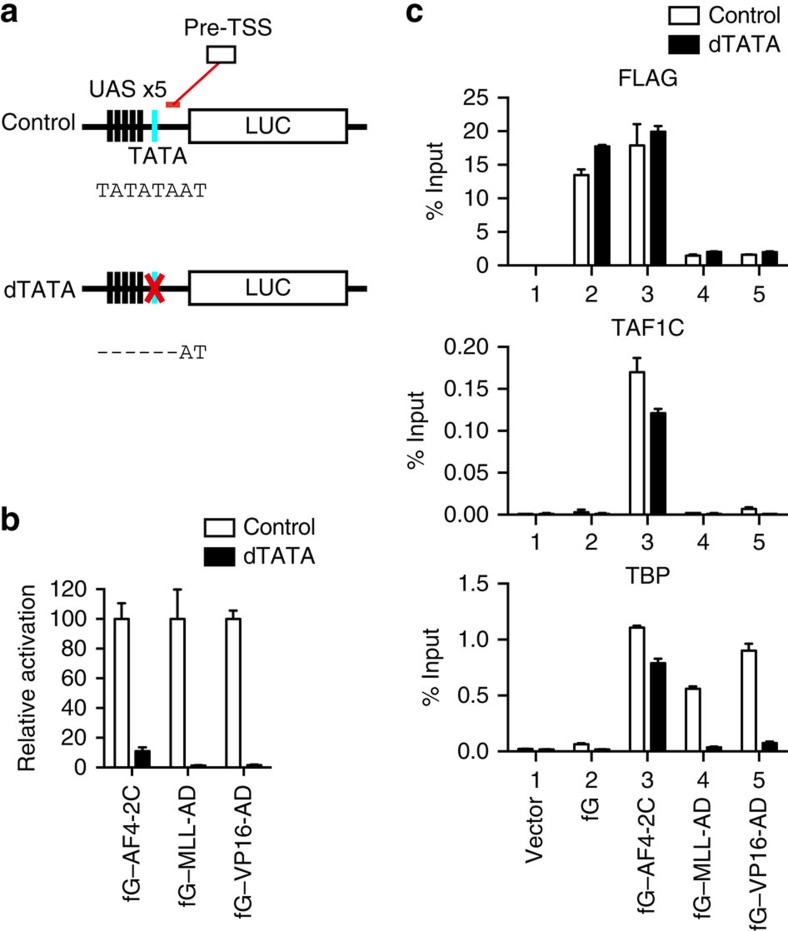
The TATA element potentiates pSER-dependent transcriptional activation. (**a**) Schematic structures of the GAL4-responsive reporter with or without the TATA element. The FR–LUC reporter is termed ‘Control' and the TATA-deficient version lacking the TATATA sequence of the TATA element is termed ‘dTATA'. (**b**) Requirement for the TATA element for transactivation by various ADs. 293T–RL–LUC cells carrying the TK–RL reporter and the FR–LUC reporter, with (control) or without (dTATA) the TATA element, were transiently transfected with the pCMV5 expression vectors for various GAL4 fusion proteins. Promoter activity was assessed with the dual luciferase reporter assay. Transcription activation activities, normalized to the RL activity, are shown relative to the value of each control (arbitrarily set as 100). (**c**) Localization of FLAG-tagged GAL4–AD fusion proteins, TAF1C and TBP on the chromatinized luciferase promoter with or without the TATA element. Each FLAG-tagged GAL4–AD fusion protein was expressed in 293T–RL–LUC (control or dTATA) cells and subjected to fanChIP–qPCR using the indicated antibodies. Precipitated DNA was analysed with qPCR, using a probe specific to the pre-transcription start site (TSS) region, as shown in **a**.

To evaluate the importance of the TATA element in pSER-dependent transactivation, we generated a reporter plasmid lacking the TATA element (Fig. 6a). The TATA-deleted reporter (dTATA) or the TATA-containing reporter (control) was stably inserted into the genome of 293T cells and the transactivation activities of fG–AF4-2C were measured. The pSER-dependent transactivation activity on the dTATA reporter was drastically impaired, compared with that on the control reporter (Fig. 6b). Similar results were obtained when reporter plasmids with or without the TATA element were transfected into 293T cells (Supplementary Fig. 5), arguing against the possibility that the observed decrease in transcription from the dTATA reporter was due to the different genomic positions of the reporter cassettes. The ADs of MLL and VP16 behaved in a similar manner (Fig. 6b and Supplementary Fig. 5), suggesting that all three transactivation modules require the presence of the TATA element for efficient transcription in this promoter context. The localization of the GAL4 fusion proteins to the GAL4-responesive elements was not affected by the absence of a TATA element (Fig. 6c). Regardless of the presence of a TATA element, the pSER domain recruited TAF1C and TBP to the promoter. On the other hand, TBP recruitment was severely impaired in MLL–AD- or VP16–AD-dependent transactivation on the dTATA reporter, suggesting that TBP was not stably tethered to the dTATA promoter as part of the TFIID complex. These results indicate that the pSER domain tethers SL1 on chromatin in a TATA element-independent manner and loads TBP onto the TATA element to initiate transcription in this promoter context.

### Functional cooperation of multiple pSER subdomains

**Figure 7:**
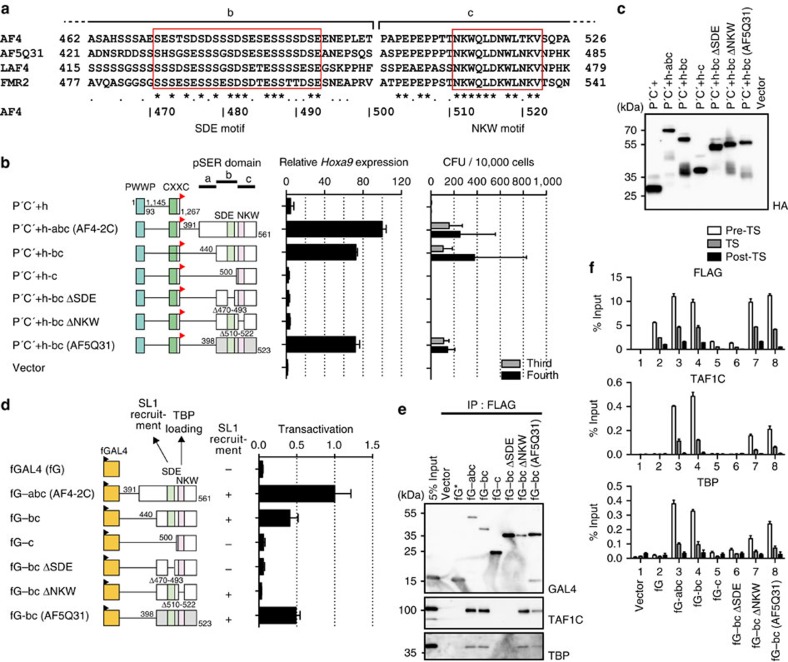
The SDE and NKW motifs collaborate to initiate transcription. (**a**) Alignment of the amino acid sequences of the SDE and NKW motifs of human AF4 family proteins. Multiple alignments were performed using the Clustal W software. ‘*', 100% amino acid identity; ‘.',75% amino acid similarity. The SDE and NKW motifs are shown in open red boxes. (**b**) Transforming ability of P′C′+ mutants fused with various pSER subdomains. *Hoxa9* expression in the first round colonies and the colony-forming units (CFUs) at the third and fourth rounds of replating are shown as described in Fig. 5a. (**c**) Protein expression of the P′C′+ mutants fused with various AF4-2C subdomains in the packaging cells. (**d**) Transactivation by various pSER subdomains. The ability to recruit SL1 is indicated. A transactivation assay was performed as described in Fig. 5c. (**e**) Association of SL1 components with various pSER subdomains. FLAG-tagged GAL4-pSER subdomains fusion proteins were expressed in 293T–LUC cells and subjected to fanChIP–WB. The sample shown in the input lane is indicated by an asterisk. (**f**) Localization of FLAG-tagged GAL4-pSER subdomain fusion proteins, TAF1C and TBP on the chromatinized luciferase promoter. Each FLAG-tagged GAL4-pSER subdomain fusion protein was expressed in 293T–LUC cells and subjected to fanChIP–qPCR as described in Fig. 5e.

**Figure 8:**
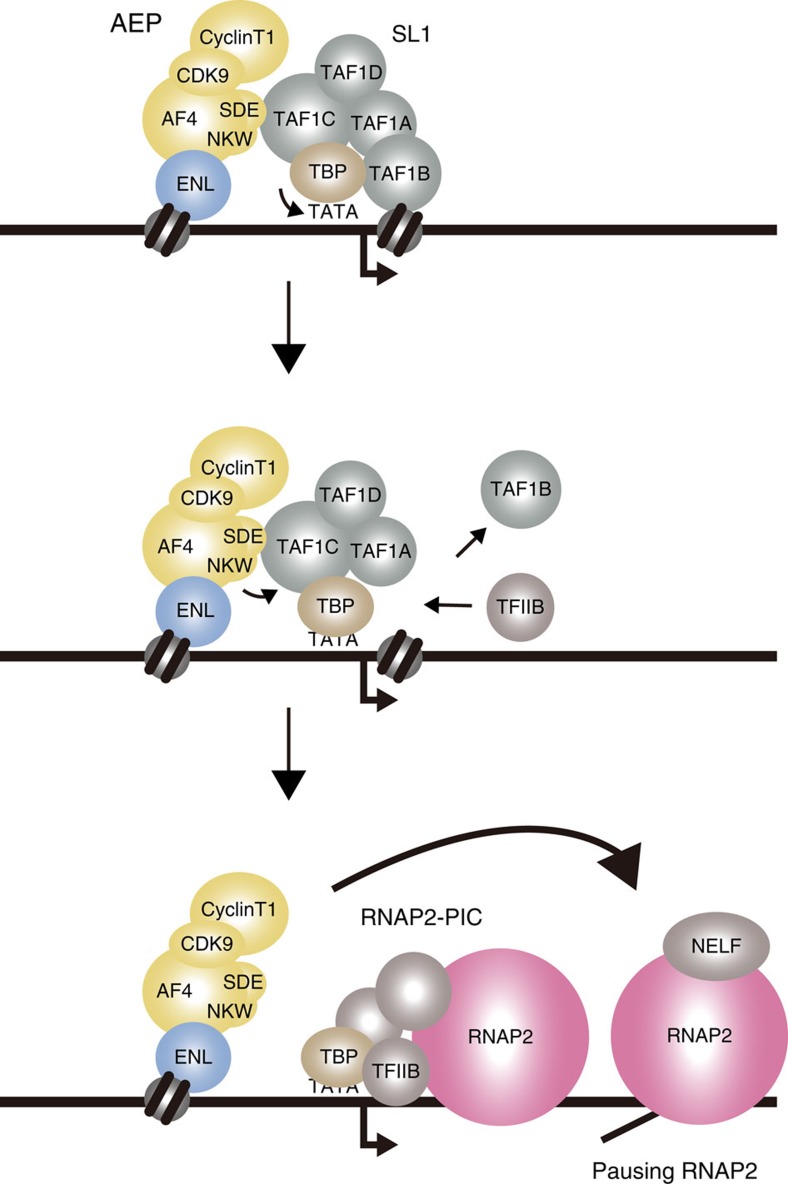
A working model. A working model of AEP-dependent transcriptional activation through SL1.

To dissect the molecular mechanism underlying transactivation by the pSER domain, we generated a series of AF4-2C mutants in which the minimum targeting module was tethered to further subdivided portions of the pSER domain (designated as a, b and c; Fig. 7a,b). The pSER domain contains two evolutionally conserved motifs, the SDE motif and the NKW motif in the b and c portions, respectively. A myeloid progenitor transformation assay showed that both the b and c portions of AF4 and AF5Q31 were required for transformation, whereas deletion of the SDE motif or the NKW motif abolished transforming ability (Fig. 7b,c). Hence, the evolutionary conserved functions of the SDE and NKW motifs are critical for MLL–AEP fusion-dependent transactivation and transformation. Consistent with these observations, a transactivation assay with the corresponding GAL4 fusion proteins showed that both the SDE and NKW motifs were necessary for transactivation (Fig. 7d). FanChIP–WB analysis showed that the SDE motif, but not the NKW motif, was sufficient for binding to SL1 (Fig. 7e). FanChIP–qPCR analysis showed that a mutant lacking the NKW motif recruited TAF1C and TBP to the promoter (Fig. 7f), suggesting that the mutant is competent in SL1 recruitment but defective in transcription initiation. Taken together, these results indicate that AF4 family proteins first recruit SL1 onto chromatin through the SDE motif and subsequently initiate RNAP2-dependent transcription through the NKW motif (Fig. 8).

## Discussion

In this study, we identified an additional important layer of complexity in the effects of MLL fusion proteins on target genes. Our results indicate that the recruitment of AF4 family proteins is a critical first step in MLL–AEP fusion-dependent leukemic transformation. The pSER domain of AF4 associated with SL1 on chromatin to activate RNAP2-dependent transcription by loading TBP onto the promoter. The results show that SL1 is a TBP-loading factor involved in gene activation induced by MLL–AEP fusion proteins and wild-type AEP.

AEP contains the P-TEFb elongation factor and associates with ELL family proteins, both of which exhibit transcription elongation activity^41^,^42^. Therefore, the AEP complex is also referred to as the super elongation complex^4^. These discoveries led to the suggestion that transcription elongation activity plays an essential role in MLL–AEP fusion-dependent leukemic transformation. However, our detailed structure/function analyses showed that neither of the binding platforms for P-TEFb or ELL conferred transforming abilities (Fig. 1b and Fig. 2c), indicating that transcription elongation activity is not sufficient for MLL–AEP fusion-dependent gene activation. Instead, the pSER domain, which harbours transcription initiation activities, conferred transforming abilities to MLL–AEP fusion proteins. Hence, the AEP complex is more than an elongation complex: it serves as a multi-functional transcriptional coactivator that facilitates many steps of the transcription cycle.

DOT1L has been shown to play important roles in MLL fusion-dependent leukemic transformation^43^. DOT1L recruitment through direct interaction enhances transforming potentials^44^. Consistent with these reports, the clonogenicities of the AF5Q31 CHD fusion proteins, which is deficient for DOT1L recruitment, were weaker than those of the ENL AHD fusion proteins (Fig. 1b,c). These results suggest a supporting role of DOT1L for AEP-dependent gene activation, which is probably mediated by its histone methyltransferease activity that establishes the chromatin environment repellent to SIRT1-dependent gene silencing^45^.

SL1, which comprises TBP and four TAF_I_ proteins, confers promoter selectivity to RNAP1 (ref. 22). On the other hand, the TFIID complex, which comprises a number of TAF_II_ proteins, plays a major role in RNAP2-depednent transcription^40^. As TBP associates with TAF_1_s and TAF_II_s in a mutually exclusive manner^19^, it has long been thought that the functions of SL1 were restricted to RNAP1-dependent transcription. However, our biochemical analyses identified SL1 as a specific binding partner of the AEP coactivator complex that activates RNAP2-dependent transcription (Fig. 2). The MLL/AEP axis maintains the expression of cellular memory genes, such as *Hox* genes (Fig. 4b,c)^3^,^46^,^47^. It is unclear how AEP/SL1-dependent transcriptional activation is advantageous in the maintenance of cellular memory genes. SL1 may have other properties in addition to promoting transcription initiation such as preventing transcriptional repression by Polycomb complexes that promote steady expression.

Our results suggest that two evolutionarily conserved motifs in the AF4 family proteins are required for the SL1-dependent activation of RNAP2-dependent transcription. One is the SDE motif, the primary binding platform for SL1. The amino acid sequence of this motif is similar to that of the SL1-binding motif in UBF^48^. Therefore, AF4–SL1 binding probably occurs through a mechanism similar to that underlying UBF–SL1 binding. The other critical motif is the NKW motif. Despite our extensive efforts to identify proteins that specifically bind this motif, none has yet been identified. Although we do not rule out the possibilities that other transcriptional regulators are recruited to the NKW motif, we infer that the NKW motif binds to the surface of SL1 itself to induce conformational changes. TAF1B is structurally similar to TFIIB^49^,^50^ and is predicted to position itself in the RNAP1–PIC, similar to TFIIB in the RNAP2–PIC^51^. Therefore, for RNAP2-dependent transcription to proceed following the binding of SL1 to the promoter, TFIIB must replace TAF1B as the binding partner of TBP. Given these observations and assumptions, we infer that (1) AF4 family proteins recruit SL1 to the target chromatin primarily through the SDE motif, (2) further association through the NKW motif changes the conformation of chromatin-bound SL1, releasing TAF1B, (3) and TAF1B-deficient SL1 is bound by TFIIB, which then forms the PIC of RNAP2 to initiate transcription (Fig. 8). The presence of a TATA element that conforms to the ∼90° bent structure required for the PIC formation^52^,^53^,^54^ potentiates this process (Fig. 6). Thus, SL1 might facilitate probing for DNA sequences suited to TBP-induced bending. SL1 might induce a similar bent structure to form the PIC of RNAP1 (ref. 51) at the ribosomal RNA promoters that lack a TATA element^22^. Most gene promoters do not contain TATA elements that satisfy the TATA box consensus sequence. It appears that TATA-like AT-rich sequences serve as alternative TBP-binding sequences to promote transcription at TATA-less promoters^55^,^56^. The AEP/SL1 complex might facilitate the formation of the TBP–DNA complex and the subsequent induction of DNA bending not only at the TATA element but also at TATA-like AT-rich sequences.

In summary, our study identified a novel cofactor of MLL–AEP fusion proteins and the AEP coactivator complex. To our surprise, the essential cofactor required for AEP-dependent gene activation is SL1, a component of the PIC of RNAP1. These results not only provide significant insights into the molecular mechanisms underlying MLL fusion-mediated leukemogenesis but also shed light on the previously unrecognized involvement of SL1 in RNAP2-dependent transcription.

## Methods

### Vector construction

The pMSCV-neo-FLAG-MLL-ENL vector and its derivatives^11^, the pBICEP2-AF4 vector^3^ and the pcDNA3.1 hygro (+)-MEN1-HA vector^12^ were generated previously. Various new gene constructs were generated through restriction enzyme digestion/PCR-based mutagenesis. The complementary DNAs were cloned into the pMSCV neo vector (for virus production; Clontech) or into the pCMV5 vector and the pcDNA4 HisMax vector (for transient expression). The pFR-LUC plasmid with the deletion of the TATATA sequence in the TATA element was made by GeneArt Gene Synthesis (Life Technologies) from the pFR-LUC plasmid (Agilent). The pLKO.1-puro-FR-LUC vectors and its blasticidin version, with or without the TATA element, were generated by restriction enzyme digestion/PCR-based mutagenesis of the pFR-LUC plasmids, the pLKO.1 vector (GE Healthcare) and a blasticidin cassette. pLKO.1-hygro–TK–RL vector and its zeocin version, which contains the Herpes simplex virus thymidine kinase promoter, the *Renilla* luciferase (RL) coding sequence and a hygromycin or zeocin cassette was generated by restriction enzyme digestion/PCR-based mutagenesis of the pRL–TK plasmid (Promega), the pLKO.1 vector and a hygromycin or zeocin cassette. The shRNA expression vectors, targeting murine *Enl* (TRCN0000084405), *Men1* (TRCN0000034394), *Mll* (TRCN0000034426), *Taf1c*#1 (TRCN0000082215) and *Taf1c*#2 (TRCN0000082217) were obtained from GE Healthcare.

### Cells and cell culture

The human leukemia cell line HB1119 (ref. 3) was cultured in RPMI 1640 medium, supplemented with 10% fetal bovine serum (FBS) and penicillin–streptomycin (PS). The 293T and 293TN (System Biosciences) cell lines and iMEFs^57^ were cultured in DMEM medium supplemented with 10% FBS and PS. To exclude the effects of the mosaic expression of *Hox* genes in iMEFs, cells were cloned once to establish a cell line that expressed a certain set of *Hox* genes homogeneously. Ecotropic virus packaging cells (PLAT-E cells)^58^ were cultured in DMEM supplemented with 10% FBS, puromycin, blasticidin and PS. 293T–LUC cells were generated by transduction of the lentivirus carrying pLKO1-puro-FR-LUC. 293T–RL–LUC cells were generated by sequential transduction of the pLKO-hygro–TK–RL reporter and the pLKO-puro-FR-LUC reporter with or without the TATA element. 293T–LUC–fG cell lines were generated by sequential transduction of pLKO-zeo–TK–RL reporter, the pLKO-bla-FR-LUC reporter and the pMSCV-hygro-fGAL4 fusion vectors.

### Antibodies

The antibodies used for the assays are described in Supplementary Table 2. The uncropped WBs are provided in Supplementary Figs 6–11.

### Virus production

The ecotropic retrovirus was produced using PLAT-E packaging cells^58^. The lentivirus was produced in 293TN cells using the pMDLg/pRRE, pRSV-rev and pMD2.G vectors^59^. The supernatant medium containing the virus was harvested 24–48 h following transfection and used in viral transduction.

### Myeloid progenitor transformation assay

The myeloid progenitor transformation assay was performed using cells harvested from the femurs and tibiae of 5-week-old female C57BL/6 mice^11^. C-Kit-positive cells were enriched using magnetic beads conjugated with an anti-c-Kit antibody (Miltenyi Biotech), transduced with a recombinant retrovirus by spinoculation and then plated in a methylcellulose medium (Iscove's modified Dulbecco's medium, 20% FBS, 1.6% methylcellulose, 100 μM β-mercaptoethanol) containing murine stem cell factors, interleukin-3 and granulocyte–macrophage colony-stimulating factors (10 ng ml^−1^ of each). G418 (1 mg ml^−1^) was added to the first round of culture to select for transduced cells. *Hoxa9* was quantified in reverse transcriptase–qPCR after the first round of culture. Colony-forming units at the third and fourth rounds were quantified per 10^4^ plated cells, after 4–6 days in culture. Experiments were approved by the Kyoto University Institutional Animal Care and Use Committee.

### Reverse transcriptase–qPCR

RNA was prepared using the RNeasy kit (Qiagen) and reverse transcribed using a Superscript III First Strand cDNA Synthesis kit, with oligo(dT) primers or random hexamers (for experiment of α-amanitin treatment; Life Technologies). Gene expression was confirmed with qPCR, using the TaqMan probes described in Supplementary Table 3 (Life Technologies). The expression levels, normalized to those of *Gapdh*, *Tbp* or *18 S rRNA*, were determined using a standard curve and the relative quantification method, as described in ABI User Bulletin #2.

### Immunoprecipitation

The expression vectors for MLL fusion proteins and the cofactors were transfected into 293T cells using the Lipofectamine 2000 reagent (Life Technologies). 293T cells cultured in a 10-cm dish were suspended in 1 ml of isotonic buffer (150 mM NaCl, 10 mM Tris-HCl pH 7.5, 1.5 mM MgCl_2_, 0.5% NP-40 and an EDTA-free protease inhibitor cocktail (Roche)). The suspension was incubated on ice for 5 min and then centrifuged at 400*g* for 3 min. The pellet was resuspended in 1 ml of lysis buffer (250 mM NaCl, 20 mM sodium phosphate pH 7.0, 30 mM sodium pyrophosphate, 5 mM EDTA, 10 mM NaF, 0.1% NP-40, 10% glycerol, 1 mM dithiothreitol (DTT) and an EDTA-free protease inhibitor cocktail) and cleared by centrifugation at 37 000*g* (R22A4; Hitachi) for 30 min at 4 °C. The supernatant was then used in IP experiments. Twenty microlitres of anti-FLAG M2 magnetic beads (Sigma) was added to each sample and the mixture was incubated for 4 h in a rotating chamber. The beads were washed five times with 500 μl of lysis buffer. The coprecipitated proteins were harvested in elution buffer (1% SDS, 50 mM NaHCO_3_). The eluted samples were mixed with an equal volume of 2 × SDS–PAGE sample buffer and then subjected to WB.

### Liquid chromatography–tandem mass spectrometry analysis

Purified proteins were visualized with Oriole staining (Bio-Rad) after SDS–PAGE analysis. The pieces of acrylamide gel containing proteins were cut out and washed with 50 mM of ammonium hydrogen carbonate containing 50% acetonitrile. The gel pieces were dried using a SpeedVac (Thermo) and suspended in 50 mM of ammonium hydrogen carbonate. The proteins were deoxidized and acetylated with the addition of 10 mM DTT and 50 mM iodoacetamide, and then digested with trypsin at 37 °C for 18 h. The digested peptides were alternately extracted in 50 mM ammonium hydrogen carbonate and acetonitrile, and then subjected to liquid chromatography–tandem mass spectrometry analysis. Peptides were separated using a NanoLC-Ultra-2D Plus system (Eksigent) and quadrupole time-of-flight mass spectrometry was performed using a Triple TOF5600 system (AB SCIEX) in an information-dependent acquisition mode. Using the acquired data sets, database searches were performed with the ProteinPilot software (AB SCIEX) and UniProtKB/Swiss-Prot database. The reliabilities of the protein identification were evaluated from the protein scores (Unused ProtScore), which were calculated using the Pro Group algorithm (AB SCIEX). Mass spectrometry was performed at the Medical Research Support Center, Graduate School of Medicine, Kyoto University.

### Transactivation assay

Transactivation assays using the pFR-LUC reporter were performed 1 day after transfection of the reporter and effector plasmids^3^. For the chromatinized templates, the expression vectors for the various GAL4 fusion proteins were transfected into 293T–LUC cells with the pRL–TK plasmid or solely transfected into 293T–RL–LUC cells. The luciferase activity was measured using a dual luciferase reporter kit (Promega). The luciferase activity values were normalized to the RL activity and expressed as the mean and s.d. of triplicate samples.

### Fractionation-assisted native chromatin IP

The chromatin fractions of the 293T, 293T–LUC, 293T–RL–LUC and HB1119 cells were prepared as follows^11^. Cells were suspended in cytoskeleton buffer (100 mM NaCl, 10 mM PIPES pH 6.8, 3 mM MgCl_2_, 1 mM EGTA pH 7.6, 0.3 M sucrose, 0.5% Triton X-100, 5 mM sodium butyrate, 0.5 mM DTT and an EDTA-free protease inhibitor cocktail) and spun down to remove the soluble fraction. The pellet was resuspended in MNase buffer (50 mM Tris-HCl pH 7.5, 4 mM MgCl_2_, 1 mM CaCl_2_, 0.3 M sucrose, 5 mM sodium butyrate, 0.5 mM DTT and a protease inhibitor cocktail) and treated with MNase. The MNase reaction was stopped by adding EDTA (pH 8.0) at a final concentration of 20 mM. Lysis buffer was then added to increase solubility. The chromatin fraction was cleared by centrifugation and immunoprecipitated with specific antibodies (Supplementary Table 2) and magnetic microbeads (Protein-G magnet beads (Invitrogen)) or with anti-FLAG M2 antibody-conjugated beads. The precipitates were washed five times with washing buffer (1:1 mixture of lysis buffer and MNase buffer with 20 mM EDTA) and then eluted in elution buffer (1% SDS and 50 mM NaHCO_3_). The eluted materials were analysed with various methods, including WB, qPCR, deep sequencing and mass spectrometry. Deep sequencing of the precipitated DNA was performed using a TruSeq ChIP Sample Prep Kit (Illumina) and Genome Analyzer IIx (Illumina) at the core facility of Hiroshima University. The data were analysed using the Integrative Genome Viewer (Broad Institute). qPCR analysis of the precipitated DNA was performed using the custom-made primer sets described in Supplementary Table 4. The value relative to the input was determined using a standard curve and the relative quantification method. Optionally, the precipitates were equilibrated with MNase buffer, treated with DNase I (Qiagen) for 10 min at 37 °C and washed five times with washing buffer to remove the DNA in the sample. The precipitates were analysed with WB or SYBR green staining.

### mRNA sequencing

Total RNA was prepared using the RNeasy kit and the quality was assessed using a eukaryote Bioanalyzer RNA Nano chip (Agilent). Deep sequencing of the total RNA was performed using SureSelect Strand Specific RNA Library Prep Kit (Agilent) and Genome Analyzer IIx (Illumina) at the core facility of Hiroshima University. Gene expression was normalized as RPKM (reads per kilo base of exon per million mapped) with the cutoff value set to 5 in the vector control. Gene set enrichment analysis was carried out using the pre-ranked method with 1,000 permutations with the gene sets. The target sets for TAF1C, ENL and MLL were defined as genes downergulated upto threefold by shRNAs transduction. The curated gene sets were obtained from Molecular signature database (MSigDB) v5.0.

### Statistical analysis

Correlation coefficient was calculated by Pearson's correlation method using GraphPad Prism 6.0 (GraphPad Software).

## Additional information

**How to cite this article:** Okuda, H. *et al*. AF4 uses the SL1 components of RNAP1 machinery to initiate MLL fusion- and AEP-dependent transcription. *Nat. Commun.* 6:8869 doi: 10.1038/ncomms9869 (2015).

## Supplementary Material

Supplementary Information Supplementary Figures 1-11 and Supplementary Tables 1-4
