# Supplementary material for: AF4 uses the SL1 components of RNAP1 machinery to initiate MLL fusion- and AEP-dependent transcription
Source: Nat Commun. 2015 Nov 23;6:8869. doi: 10.1038/ncomms9869 (PMC4673504; doi:10.1038/ncomms9869)
Supplement: Supplementary Information — Supplementary Figures 1-11 and Supplementary Tables 1-4 [file ncomms9869-s1.pdf]

Supplementary Figures

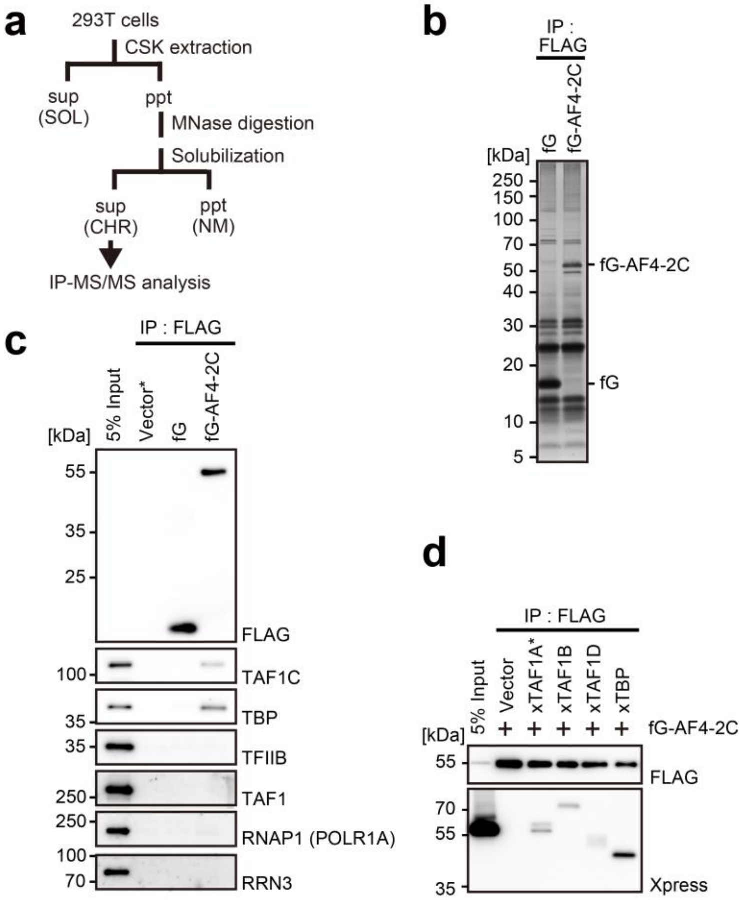

Supplementary Figure 1 The pSER domain associates specifically with the SL1 complex. (a) Schema of fanChIP analysis. CSK: cytoskeleton buffer; MNase: micrococcal nuclease; sup: supernatant; ppt: pellet; SOL: soluble fraction; CHR: chromatin fraction; NM: nuclear matrix fraction (b) An Oriole-stained image of proteins from 293T cells transiently expressing fG-AF4-2C. Protein was purified using the fanChIP method. (c) The pSER domain specifically associates with endogenous SL1 components. 293T cells were transiently transfected with expression vectors for fG-AF4-2C or fG. FanChIP analysis of the chromatin fraction was performed using an anti-FLAG antibody. FLAG-tagged GAL4 fusion proteins and various endogenous proteins were visualized using the antibodies indicated. The sample shown in the input lane is indicated by an asterisk. (d) Association of SL1 components with the pSER domain. Xpress-tagged SL1 components (xTAF1A, xTAF1B, xTAF1D, and xTBP) were co-expressed with fG-AF4-2C in 293T cells and subjected to fanChIP-WB.

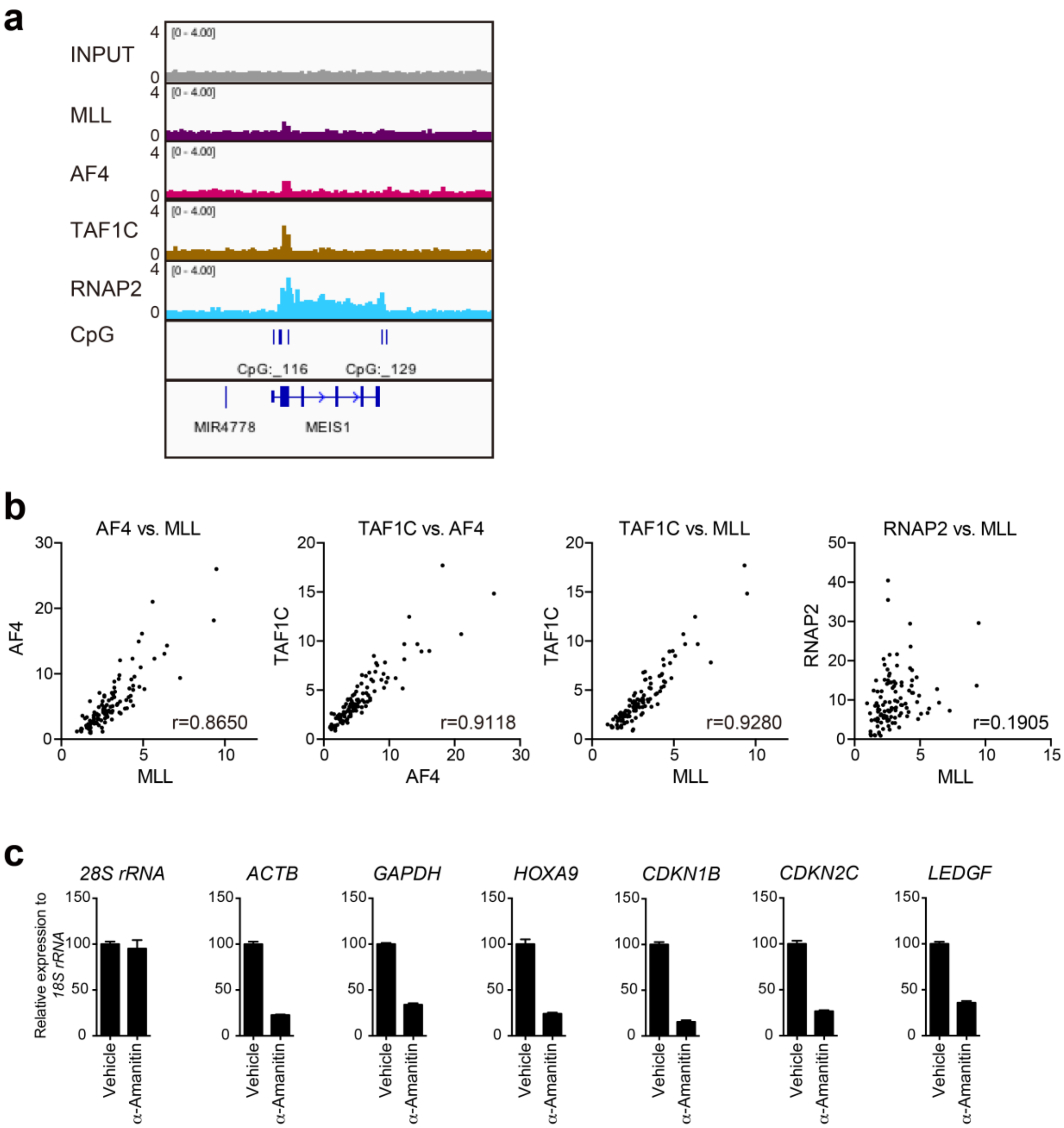

Supplementary Figure 2 Correlation of ChIP signal intensities at the promoter regions of MLL target genes and effect of α-amanitin on MLL target gene expression. (a) Localization of MLL-ENL, AF4, TAF1C, and RNAP2 at the MEIS1 locus in HB1119 cells. Chromatin from HB1119 cells was subjected to fanChIP-seq using the indicated antibodies, as described for Figure 3a. (b) Correlation between the ChIP signals of various proteins at the promoter-proximal regions of MLL target genes. FanChIP-seq analyses of HB1119 cells were performed, as described for Figure 3a. ChIP-seq tags were clustered into a 3-kb bin (0 to 3 kb from the TSS). The ChIP signal intensities of the indicated proteins at each MLL target gene were plotted. One data point fell

outside the axis limits in the “MLL vs. RNAP2” plot. The Pearson correlation coefficients are shown in the plots. (c) Effect of α-amanitin on MLL target gene expression. 10 μg ml^{-1} of alpha-amanitin solubilized in PBS was added to HB1119 cells for 24 h, and the expression of MLL target genes was analyzed with RT-qPCR. The expression level of each gene was normalized to that of 18S rRNA. The expression level is shown relative to the value of the control vector (arbitrarily set at 100%) with error bars (SD of PCR triplicates).

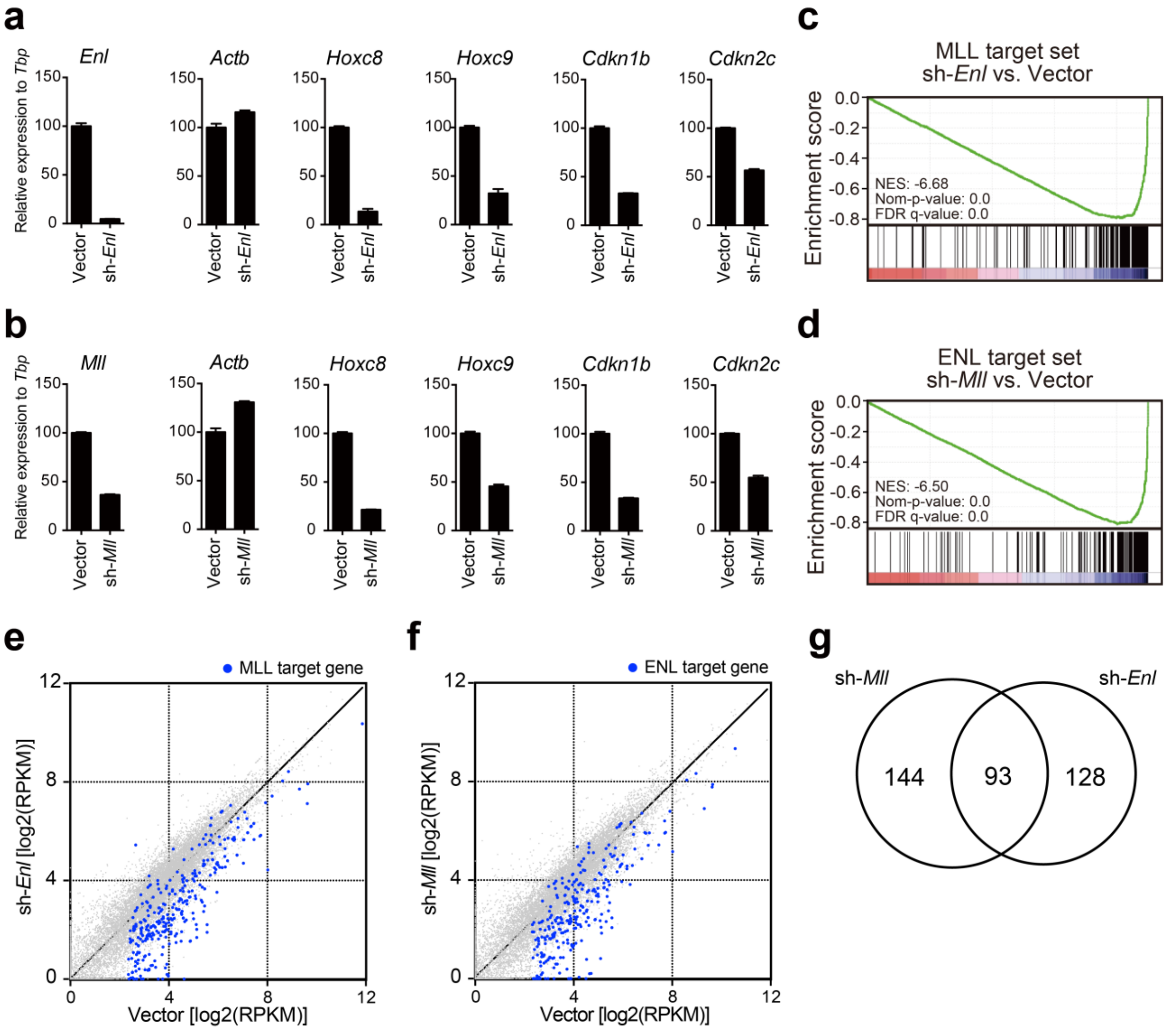

Supplementary Figure 3 Effect of *Taf1c*, *Enl* and *Mll* knockdown on gene expression. (a) Effect of *Enl* knockdown on AEP-dependent gene activation. *Enl* was knocked down with shRNA in iMEFs, and the expression of AEP target genes was analyzed with RT-qPCR, as described for Figure 4b. The expression of each gene was normalized to that of *Tbp*. The expression level is shown relative to the value of the control vector (arbitrarily set at 100%) with error bars (SD of PCR triplicates). (b) Effect of *Mll* knockdown on AEP-dependent gene activation. *Mll* was knocked down with shRNA in iMEFs, and the expression of AEP target genes was analyzed with RT-qPCR, as in (a). (c) GSEA of the expression profiles of iMEFs, with or without *Enl* knockdown. Genes that exhibited a greater than three-fold decrease upon *Mll* knockdown in RNA-seq analysis were defined as “MLL target genes.” The MLL target gene set was downregulated by *Enl* knockdown. (d) GSEA of the expression profiles of iMEFs, with or

without *Mll* knockdown. Genes that exhibited a greater than three-fold decrease upon *Enl* knockdown in RNA-seq analysis were defined as “ENL target genes.” The ENL target gene set was downregulated by *Mll* knockdown. (e) Scatter plot of gene expression levels with or without *Enl* knockdown. MLL target genes are highlighted in blue. (f) Scatter plot of expression levels with or without *Mll* knockdown. ENL target genes are highlighted in blue. (g) Overlap diagram of the number of down-regulated genes by *Enl* and *Mll* knockdown.

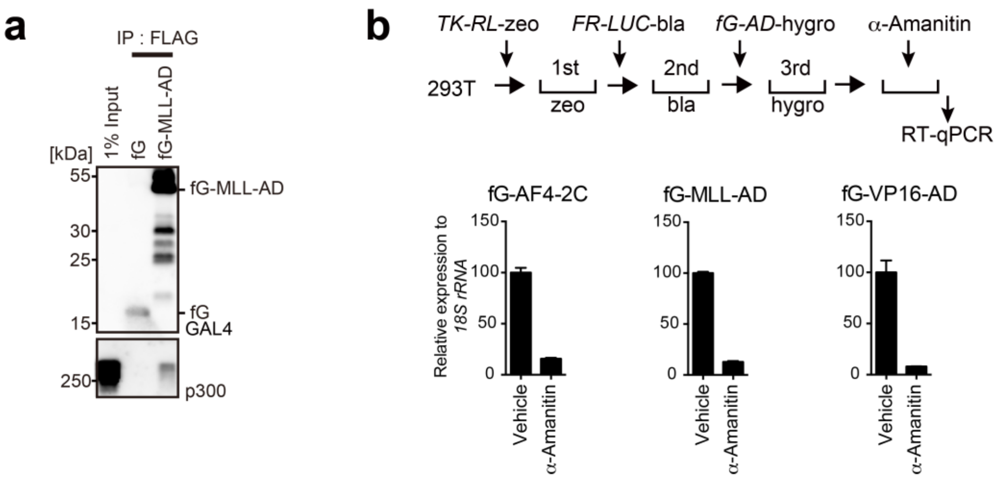

Supplementary Figure 4 Biological properties of various transactivation domains (a) The activation domain (AD) of MLL associates with p300. 293T cells were transiently transfected with the expression vector for the FLAG-tagged GAL4-MLL-AD protein (fG-MLL-AD). FanChIP-WB analysis was performed using an anti-FLAG antibody. The GAL4 fusion proteins and endogenous p300 proteins were visualized using an anti-GAL4 antibody and an anti-p300 antibody, respectively. (b) Effect of α-amanitin on transactivation activity of various transactivation domains. 293T-LUC-fG cells were generated by sequential transduction of the pLKO-zeo-TK-RL reporter, pLKO-bla-FR-LUC reporter and the pMSCV-hygro-fGAL4-AD. 10 μg ml^{-1} of α -amanitin solubilized in PBS was treated for 24 h and the expression of MLL target genes was analyzed with RT-qPCR. The expression of firefly luciferase was normalized to that of 18S rRNA. The expression level is shown relative to the value of the PBS treatment (arbitrarily set at 100%) with error bars (SD of PCR triplicates). Bla: blasticidin; zeo: zeocin; hygro: hygromycin

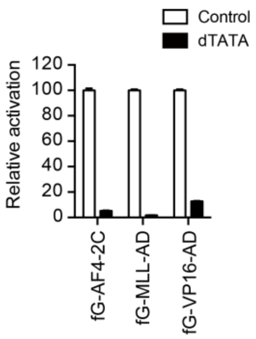

Supplementary Figure 5 Transactivation activities of various transactivation domains (ADs) on naked reporter plasmids with or without the TATA element. Transcription activation activity of various GAL4-AD fusion proteins on the naked reporter plasmid. 293T cells were transiently transfected with expression vectors for various FLAG-tagged GAL4 fusion proteins, the pRL-TK reporter plasmid, and the pFR-LUC reporter plasmid with (Control) or without the TATA element (dTATA). Promoter activity was assessed with the dual luciferase reporter assay. The normalized transcription activation activity is shown relative to the value of each control (arbitrarily set at 100).

Original western blot data for Fig. 1c

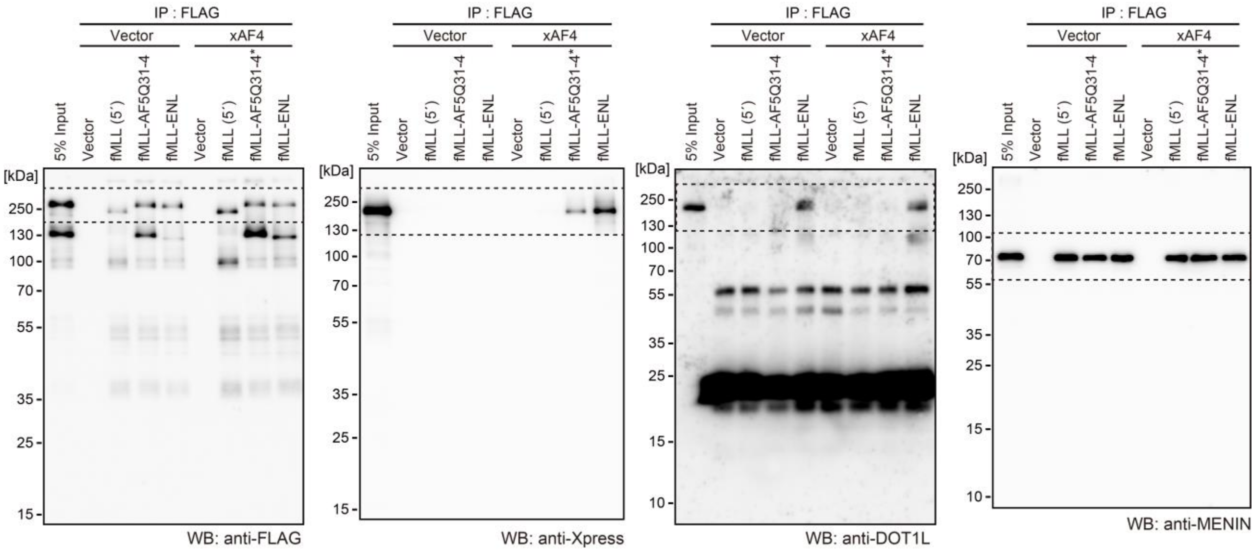

Original western blot data for Fig. 1d

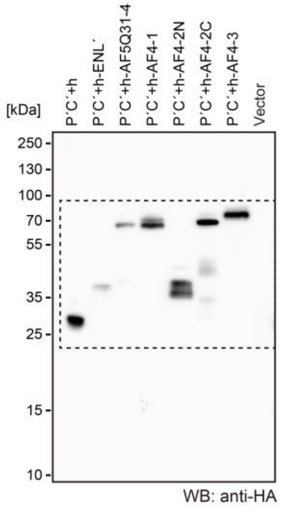

Supplementary Figure 6 Original western blot data for Figure 1c and 1d

Original western blot data for Fig. 2c

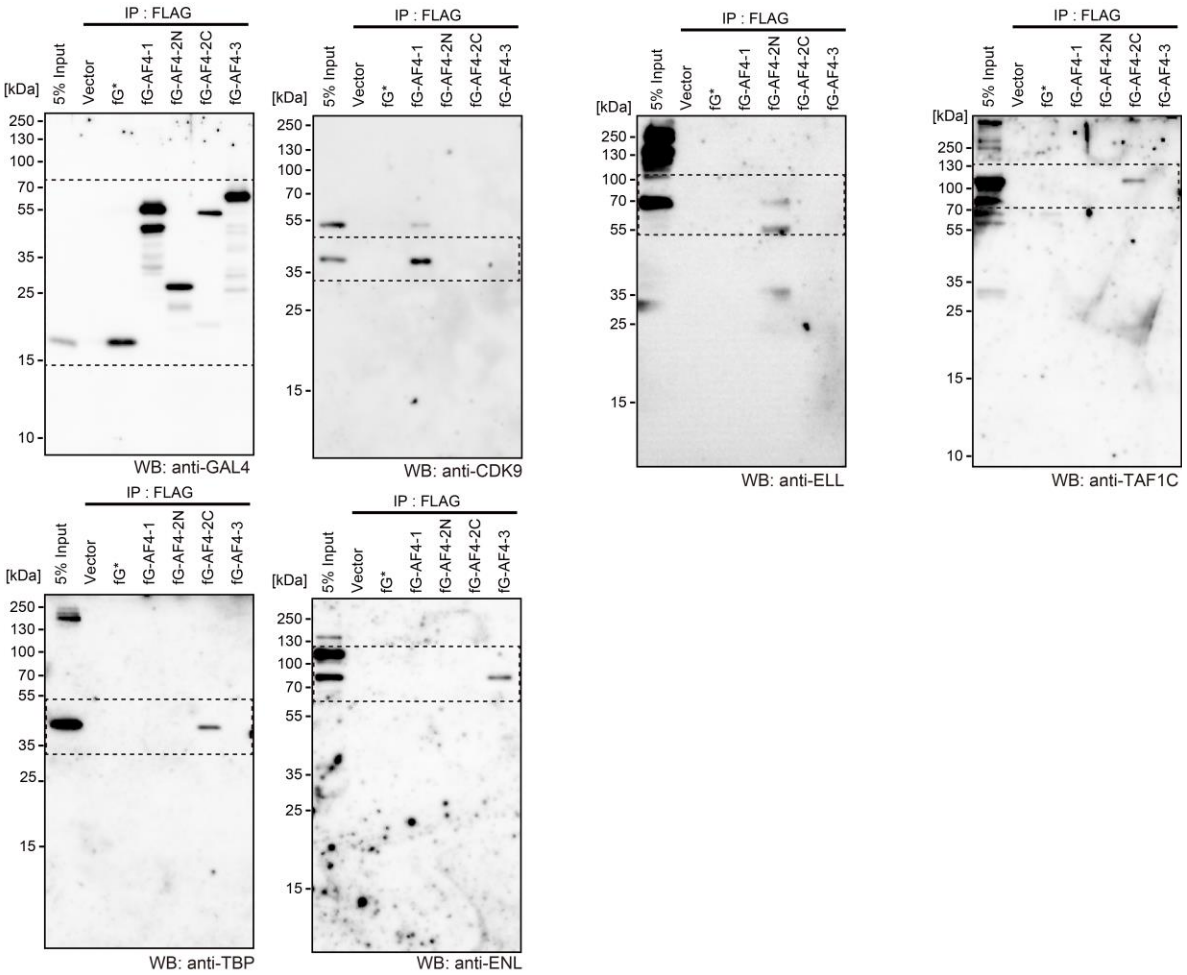

Original western blot data for Fig. 2d

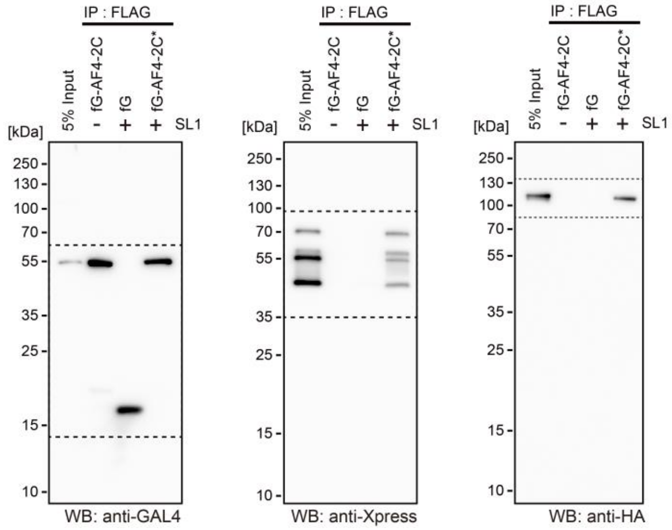

Supplementary Figure 7 Original western blot data for Figure 2c and 2d

Original western blot data for Fig. 2e

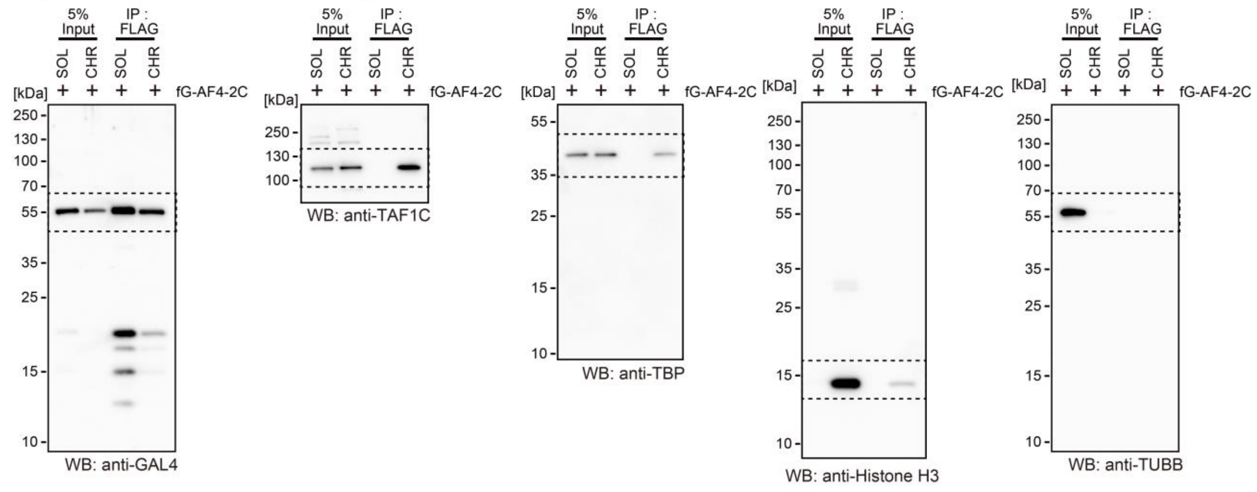

Original western blot and SYBR green stain data for Fig. 2f

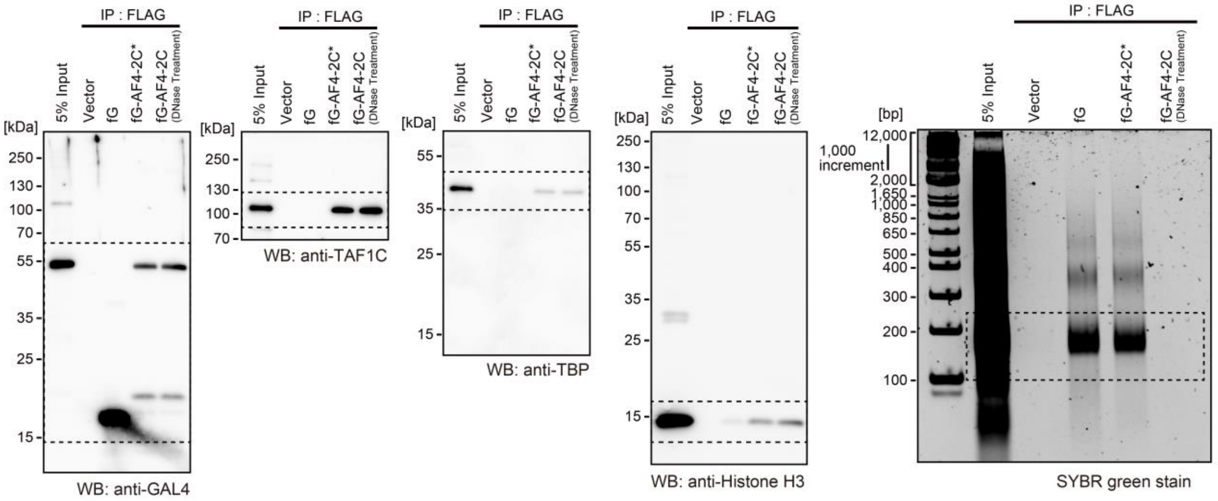

Supplementary Figure 8 Original western blot data and gel image for Figure 2e and 2f

Original western blot data for Fig. 2g

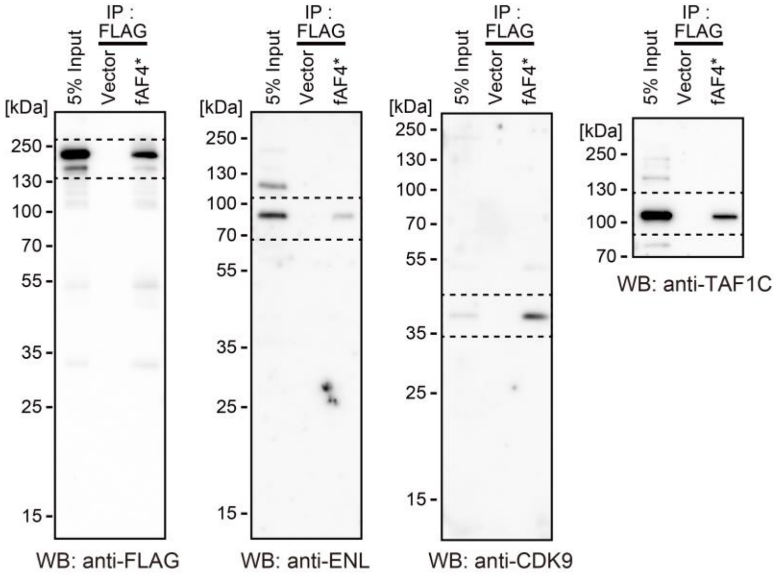

Original western blot data for Fig. 2h

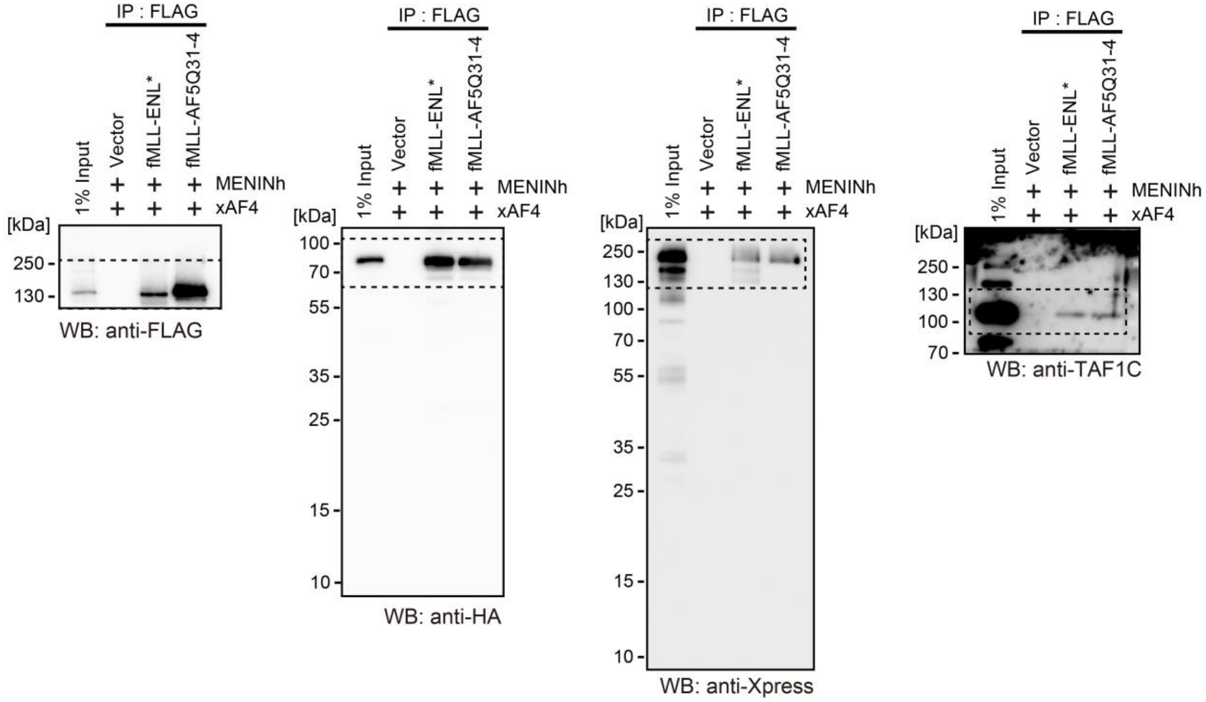

Supplementary Figure 9 Original western blot data and gel image for Figure 2g and 2h

Original western blot data for Fig. 5b

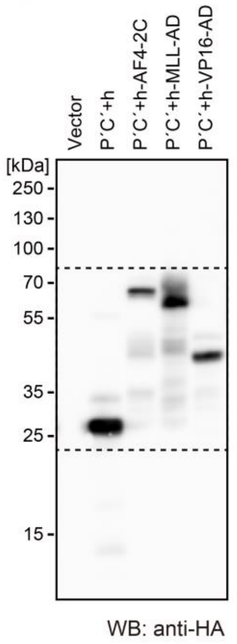

Original western blot data for Fig. 5d

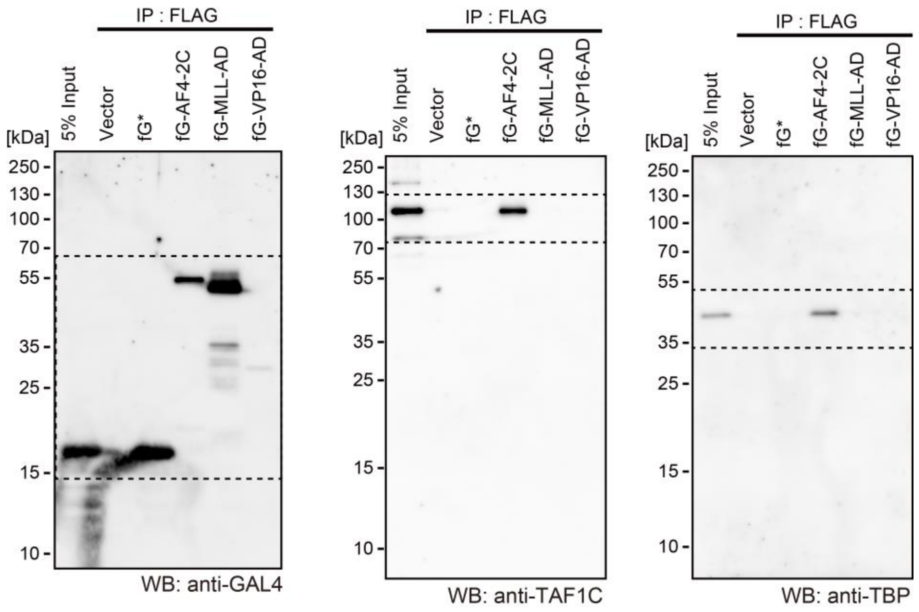

Supplementary Figure 10 Original western blot data and gel image for Figure 5b and 5d

Original western blot data for Fig. 7c

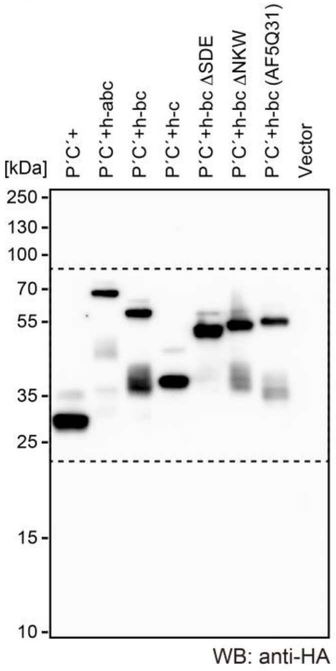

Original western blot data for Fig. 7e

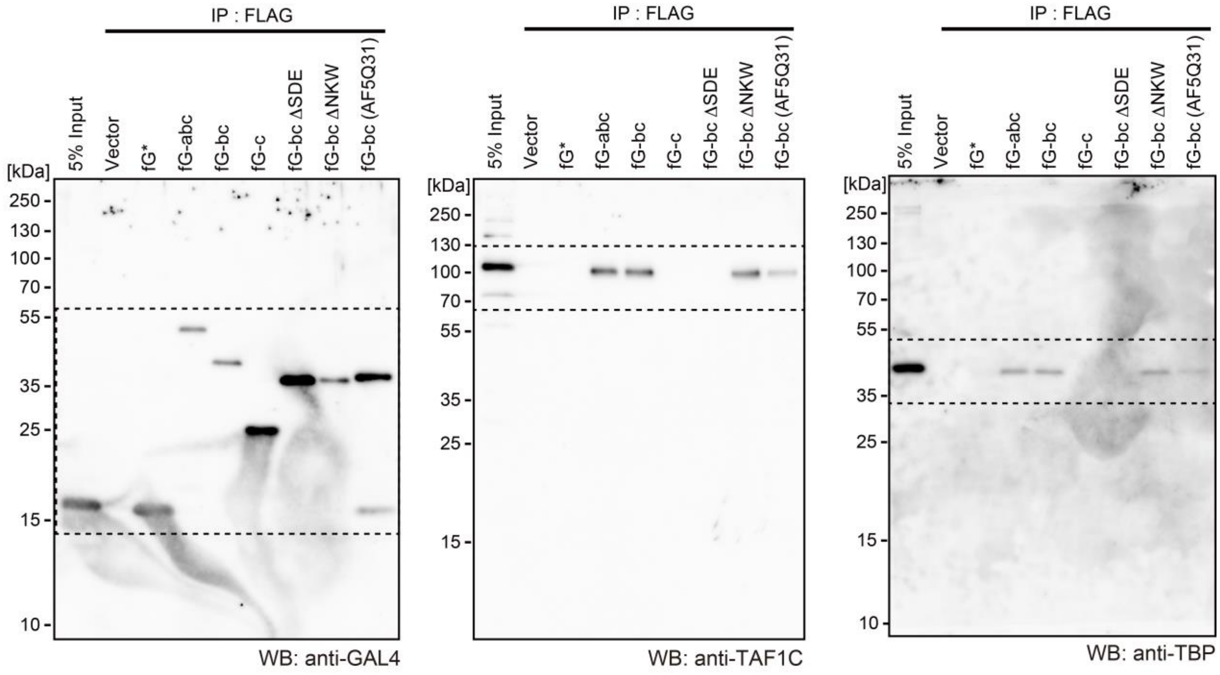

Supplementary Figure 11 Original western blot data and gel image for Figure 7c and 7e

Supplementary Tables

Supplementary Table 1. List of MLL target genes in HB1119 cells

ACTB	C20orf103	FAM36A	LOC401093	PAX5	SMAD3	ZEB2
ACTG1	CCND3	FLT3	LOC401097	PBX3	SMC4	ZFHX3
ADRBK1	CD7	GABPB1	LRRK1	PCBP1	SNHG7	ZFP36L2
AKAP8	CDCA7	GPR150	MACF1	PIM3	SOCS2	ZMYND11
ANKRD18B	CDK6	GTF2A1	MAZ	PLEKHO1	SOX4	
ANP32A	CDKN1B	GUCY1A3	MBNL1	PROM1	SRSF7	
ANP32B	CDKN2C	H2AFX	MEF2A	PTBP1	SUPT3H	
APOLD1	CHSY1	H2AFY	MEIS1	PTMA	SUV420H1	
ARID1A	COMMD3	H6PD	MFSD11	RASGRF1	TAPT1	
ARID1B	CPEB2	HMGB1	MID1IP1	RCC1	TBC1D14	
ASF1A	CTBP1	HNRNPAB	MIR17HG	REEP3	TCF12	
BAHCC1	DLEU1	HNRNPU	MRPS34	RHOBTB3	TMSB4X	
BAZ1A	DLEU2	HOXA10	MYB	RNF220	TNRC18	
BCL11A	EEF1A1	HOXA9	MYC	RPL10A	TPM4	
BCL7A	EIF4A1	IRF2BP2	NAT10	RUNX2	TRA2B	
BMI1	EIF4A2	IRX3	NR3C1	RXRA	UBC	
BZRAP1-AS1	EPHA8	JMJD1C	OAZ1	SENP6	UBE2QL1	
C18orf25	FAM108C1	LMNB1	OTUD1	SFPQ	ZC3H12C	

Supplementary Table 2. Antibodies used in this study

Antigen	Antibody type	ID/product no.	Source/reference	Application (Dilution)
FLAG tag	Rabbit polyclonal	F-7425	Sigma	WB (1/1,000)
FLAG tag	Mouse monoclonal	F-3165	Sigma	ChIP
HA tag	Rat monoclonal	3F10	Roche	WB (1/1,000)
Xpress	Rabbit polyclonal	sc-499	Santa Cruz Biotech.	WB (1/1,000)
GAL4	Rabbit polyclonal	sc-577	Santa Cruz Biotech.	WB (1/1,000)
TAF1C	Rabbit polyclonal	A303-698A	Bethyl Laboratories	WB (1/1,000)
TAF1C	Rabbit polyclonal	ab134394	Abcam	ChIP
TBP	Rabbit polyclonal	sc-204	Santa Cruz Biotech.	ChIP, WB (1/200)
Histone H3	Rabbit polyclonal	39163	Active Motif	WB (1/2,000)
Tubulin, beta	Rabbit polyclonal	ab6046	Abcam	WB (1/1,000)
CDK9	Rabbit polyclonal	sc-8338	Santa Cruz Biotech.	WB (1/200)
ELL	Rabbit polyclonal	A301-645A	Bethyl Laboratories	WB (1/1,000)
ENL	Rabbit polyclonal	A302-267A	Bethyl Laboratories	WB (1/1,000)
MLL	Rabbit polyclonal	rpN1	Yokoyama et al. 2005 Cell 123, 207–18	ChIP
MENIN	Rabbit polyclonal	A300-105A	Bethyl Laboratories	ChIP, WB (1/1,000)
DOT1L	Mouse monoclonal	sc-390879	Santa Cruz Biotech.	WB (1/200)
AF4	Goat polyclonal	sc-49350	Santa Cruz Biotech.	ChIP
NELF-A	Rabbit polyclonal	A301-910A	Bethyl Laboratories	ChIP
CDK9	Rabbit monoclonal	ab76320	Abcam	ChIP
RNAP2	Mouse monoclonal	05-263	Millipore	ChIP
TAF1	Mouse monoclonal	sc-393981	Santa Cruz Biotech.	ChIP
TAF1	Rabbit polyclonal	A303-505A	Bethyl Laboratories	WB (1/1,000)
TFIIB	Rabbit polyclonal	sc-225	Santa Cruz Biotech.	WB (1/200)
RNAP1(POLR1)	Mouse monoclonal	sc-48385	Santa Cruz Biotech.	WB (1/200)
RRN3	Rabbit polyclonal	ab112052	Abcam	WB (1/1,000)
p300	Rabbit polyclonal	A300-358A	Bethyl Laboratories	WB (1/1,000)

Supplementary Table 3. Primers used for RT-qPCR

TaqMan Probes for mouse transcripts

Gene	Probe ID
Gapdh	Mm99999915_g1
Actb	Mm00607939_s1
Tbp	Mm00446971_m1
Taf1c	Mm00498790_m1
Enl (Mllt1)	Mm00452080_m1
Mll (Mll1)	Mm01179235_m1
Hoxa9	Mm00439364_m1
Hoxa10	Mm00433966_m1
Hoxc8	Mm00439369_m1
Hoxc9	Mm00433972_m1
Cdkn1b	Mm00438168_m1
Cdkn2c	Mm00483243_m1
Runx2	Mm00501580_m1
Af4 (Aff1)	Mm00547601_s1

TaqMan Probes for human transcripts

Gene	Probe ID
ACTB	Hs99999903_m1
GAPDH	Hs02758991_g1
HOXA9	Hs00365956_m1
CDKN1B	Hs01597588_m1
CDKN2C	Hs00176227_m1
LEDGF (PSIP1)	Hs00253515_m1

Custom TaqMan Probes for human transcripts

Probes	Forward primer seq.	Reverse primer seq.	Reporter seq.
18S rRNA	GCAGGCGCGGGTAAC	AAGCTTATGACCCGCACTTACTC	CCGTTGAACCCCATTCGT
28S rRNA	AGTACGAATACAGACCGTGAAAGC	TCTGACACCTCCTGCTTAAAACC	ACGATCCTTCTGACCTTTT
FR LUC	CATCCGGTTTTGGAATGTTTACTACA	GGGATCGTAAAAACAGCTCTTCTTCA	ACGACTCGAAATCCAC

Supplementary Table 4. Primers used for ChIP-qPCR

Probes	Forward primer seq.	Reverse primer seq.	Reporter seq.
ARP1 pre-TSS	AGGGCAGTTGCTCTGAAGTC	CTGCAGAAGGAGCTCTTGGA	ACTGCCTGGCCACTCC
ARP1 TSS	AGAGAGAGTGCGAGACCGA	GCCACTGGCAGTTTCTTTCTG	CCTCTCCAGCTTTCTC
ARP1 post-TSS	CAGGCCCAAGCGAATTACCT	AGTTGACTGGTGATCAATTTAAAGGAGTT	CTGGATGCCAAGCTCT
HOXA7 pre-TSS	GCCTTCCCCGTCTGGAT	ACTCTGCCCAAGTCTTCTCTCA	CAGGCCGGACTTAGAC
HOXA7 TSS	GACGCCTACGGCAACCT	GCCTTTGGCGAGGTCACT	CCCTGCGCCTCCTAC
HOXA7 post-TSS	TGCCAGGGTCCATTTCAAGATG	CCCTCATCCCCAGGACCTT	CTCTGTCCTCATTCCC
HOXA9 pre-TSS	TGGCTGCTTTTTTATGGCTTCAATT	CCGCGTGCGAGTGC	CCCCTCACATAAAATT
HOXA9 TSS	TCACCACCACCCCTACGT	GCAAGCCCGCGAAGGA	CAGGAGCGCATGTACC
HOXA9 post-TSS	AGTGGCGGCGTAAATCCT	TGATCACGTCTGTGGCTTATTTGAA	CCCGCAGCCTCATC
CDKN1B pre-TSS	GTCCCGAGGGTCCCTTC	GTGTGCCTACCTCATCTCATACG	CAGCTGTCACATTCTG
CDKN1B TSS	GGGTCTGTGTCTTTTGGCT	GCCCGAACCCCTCTCG	CCAGCGACTGCCCTC
CDKN1B post-TSS	GCTTTGGGAGAGCTAACTTTATTGGT	CGGATCTTACCATCTCCAGTTTCTG	ACCTGGCCCACTGCTT
CDKN2C pre-TSS	CTCCACAACCGTCTTAAATAACAAACC	GCGGGCTTGAGTCTGTGA	CAGCTGCCCCAATTC
CDKN2C TSS	GGCGGCTGCCCTGT	CCCGGTGCCACTTTGC	CTGTGCCCCTTTGCTG
CDKN2C post-TSS	CTGTGGAGTCGTCAGAATTCTTCAT	CGATTCACACGTGATTATTCAGCAAA	CCTCGCCTCGCTTTT
LEDGF pre-TSS	CCACCTACCAGCTCCTATTCTACTA	GGATGTGAGTTTGGGCCCTAA	TAGCTGCATCTAAATTTT
LEDGF TSS	CCCCGGCAGGTGAGC	GCCCAGCGGCTGCA	TTCCCCGCTACAGCCAG
LEDGF post-TSS	TGTTTAAAAATTAGTGAAACATTGACATTTCCATAGTT	TTCTCTGACATCCAAGTGTTTGTGT	TTGGATCAAGTACAAAATATC
LUC pre-TSS	CGGCGCCATTCTATCCTCTAG	AGGGCGTATCTCTTCATAGCCTTAT	CTCCAGCGGTTCCATC
LUC TSS	GGGCTGAATACAAATCACAGAATCG	CCAACACCGGCATAAAGAATTGAAG	ATGCAGTGAAAACTCT
LUC post-TSS	CATCACGGTTTTGGAATGTTTACTACA	GGGATCGTAAAAACAGCTCTTCTTCA	ACGACTCGAAATCCAC
